# Organic-to-inorganic structural chirality transfer in a 2D hybrid perovskite and impact on Rashba-Dresselhaus spin-orbit coupling

**DOI:** 10.1038/s41467-020-18485-7

**Published:** 2020-09-17

**Authors:** Manoj K. Jana, Ruyi Song, Haoliang Liu, Dipak Raj Khanal, Svenja M. Janke, Rundong Zhao, Chi Liu, Z. Valy Vardeny, Volker Blum, David B. Mitzi

**Affiliations:** 1 Department of Mechanical Engineering and Materials Science, Duke University, Durham, NC 27708 USA; 2 Department of Chemistry, Duke University, Durham, NC 27708 USA; 3 Department of Physics and Astronomy, University of Utah, Salt Lake City, UT 84112 USA

**Keywords:** Optical materials, Two-dimensional materials, Spintronics, Electronic structure

## Abstract

Translation of chirality and asymmetry across structural motifs and length scales plays a fundamental role in nature, enabling unique functionalities in contexts ranging from biological systems to synthetic materials. Here, we introduce a structural chirality transfer across the organic–inorganic interface in two-dimensional hybrid perovskites using appropriate chiral organic cations. The preferred molecular configuration of the chiral spacer cations, *R*-(+)- or *S*-(−)-1-(1-naphthyl)ethylammonium and their asymmetric hydrogen-bonding interactions with lead bromide-based layers cause symmetry-breaking helical distortions in the inorganic layers, otherwise absent when employing a racemic mixture of organic spacers. First-principles modeling predicts a substantial bulk Rashba-Dresselhaus spin-splitting in the inorganic-derived conduction band with opposite spin textures between *R*- and *S*-hybrids due to the broken inversion symmetry and strong spin-orbit coupling. The ability to break symmetry using chirality transfer from one structural unit to another provides a synthetic design paradigm for emergent properties, including Rashba-Dresselhaus spin-polarization for hybrid perovskite spintronics and related applications.

## Abstract (web summary)

Inversion asymmetry imparts rich condensed matter phenomena in inorganic systems, and transmission of chirality across structural motifs is an attractive design strategy to break symmetry. Here, the authors use chiral organic cations to transfer structural chirality to inorganic layers in hybrid perovskites.

## Introduction

Inversion asymmetry is at the heart of various physical properties of inorganic systems. Especially in conjunction with relativistic effects such as spin–orbit coupling (SOC), it engenders rich condensed matter phenomena, such as the quantum spin Hall effect^1^, topological surface states, and Rashba/Dresselhaus coupling^2–4^ in non-magnetic systems as well as exotic spin topologies including chiral domain walls^5^ and skyrmions^6^ in magnetic systems, with promising applications in electronic, magnetic, and spintronic technologies^7,8^. One potential route for inducing asymmetry relies on chirality, a fundamental design feature based on lack of inversion and mirror symmetries that permeates all hierarchies of molecular organization and assembly^9^. Transmission of chiral information and asymmetry across structural motifs and length scales has a vital role in biological as well as synthetic systems. The chirality transfer is often expressed as cooperative self-assembly of chiral organic monomers into supramolecular^10^ and macroscopic chiral aggregates^11^ or templated chiral helicity in otherwise achiral molecules^12^ through intra- and inter-molecular interactions. While such chirality transfer phenomena have been mainly established in organic systems, the ability to exploit the influence of chiral organic molecules to structurally modify an extended inorganic lattice and thereby engender emergent properties based on crystal asymmetry remains underexplored.

In this context, two-dimensional (2D) layered hybrid organic–inorganic perovskites (HOIPs) provide ideal platforms to study structural chirality transfer across organic–inorganic interfaces. They exhibit crystallographically well-ordered structures with alternating organic and inorganic layers, coupled with broad flexibility in the choice of organic cations and inherent sensitivity of properties to structural tuning. 2D HOIPs are currently in the spotlight owing to their chemical stability, structural versatility as well as exceptional photophysical properties, including large exciton binding energies, high photoluminescence quantum efficiency, strong exciton–phonon couplings and tailorable optoelectronic properties for light-emitting and energy-related applications^13–15^. Chiral organic cations have been recently employed in 2D HOIPs, resulting in ferroelectricity^16^ and chiroptical properties such as circular dichroism and circularly polarized luminescence^17,18^. Notably, the inorganic-related chiroptical activity in these already reported HOIP systems is evidently an optically induced phenomenon caused by dipolar interactions with chiral cations^19,20^, and thus, the chain of evidence for structural chirality/asymmetry transfer to the inorganic layers remains incomplete if solely based on chiroptical spectroscopies. Indeed, we find (as will be discussed later) that the introduction of a chiral organic cation in 2D HOIPs does not necessarily imply a significant degree of structural chirality within the inorganic framework.

Here, we demonstrate a structural chirality transfer across the organic–inorganic interface in a prototypical 2D HOIP, *R*-(+)- or *S*-(−)-1-(1-naphthyl)ethylammonium lead bromide, wherein the enantiopure chiral spacers induce symmetry-breaking helical distortions in the inorganic framework via asymmetric hydrogen-bonding interactions, otherwise absent when employing a racemic mixture of organic spacers. As a result, the *P*2_1_ chiral symmetry of the organic sublattice “transfers” to the inorganic sublattice. The use of chiral organic spacers imparts added functionalities to the semiconducting inorganic framework including (a) distinct circular dichroism (CD) of excitonic absorption in the [PbBr_4_]^2−^ layers, induced by net crystallographic handedness and dipolar interactions with polarizable $\pi$ clouds of chiral organic cations, and (b) Rashba–Dresselhaus (RD) spin-splitting of otherwise two-fold spin-degenerate electronic bands as a consequence of the broken inversion symmetry (resulting from organic-to-inorganic chirality transfer) and strong SOC. While the chiroptical activity enables detection of circularly polarized light in practical applications^21^, the RD effect (the focus of the current study) is actively pursued in the field of spintronics^8,22,23^, which relies on the spin degrees of freedom. In the present chiral HOIP, hybrid density-functional theory (DFT) calculations are used to predict a substantial bulk RD splitting of the inorganic-derived conduction band, which is absent in the racemic analog.

## Results

### Structural chirality transfer in 2D hybrid organic–inorganic perovskites

**Fig. 1:**
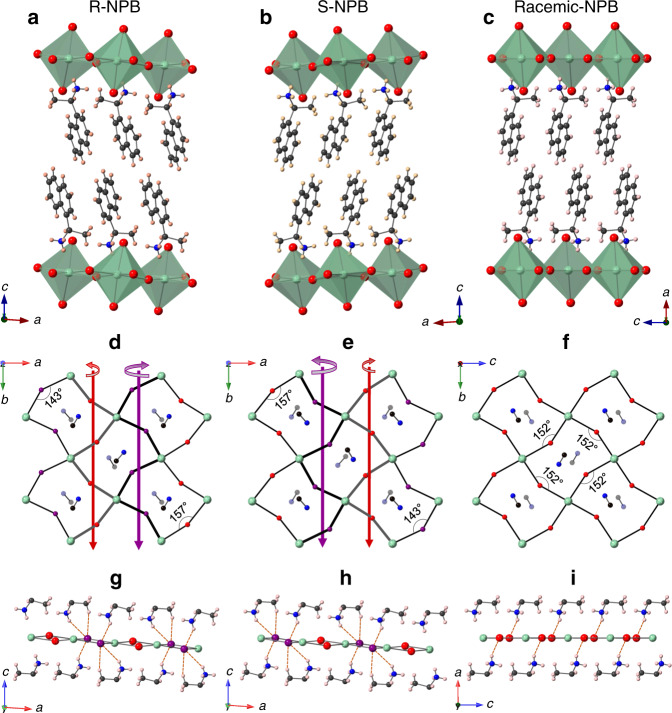
Structural characteristics of chiral- and racemic-NPB. **a**–**c** Schematic X-ray single-crystal structures of **a** *R*-NPB, **b** *S*-NPB, and **c** racemic-NPB. **d**, **e** In-plane views of [PbBr_4_]^2−^ layers in **d** *R*-NPB and **e** *S*-NPB show two different equatorial Pb–Br–Pb bond angles of 143° and 157° associated with two different Br atoms, denoted as purple and red spheres, respectively. The axial Br atoms are omitted for clarity. Opposite and dissimilar out-of-plane helical distortions (indicated by small and big curved arrows) of red and purple Br atoms can be seen from the spiraling of corresponding Pb–Br–Pb bonds (marked by thicker black and gray lines in **d** and **e**) around the 2_1_-screw axes (red and purple arrows). **f** Racemic-NPB shows a single equatorial Pb–Br–Pb bond angle of 152° with no out-of-plane distortions of Br atoms. Also shown in **d**–**f** are the organic terminal -CH-NH_3_^+^ groups, represented as solid and shaded dumbbells for the upper and lower organic layers, respectively. **g**–**i**, Hydrogen-bonding interactions between the equatorial Br atoms and NEA^+^ cations in **g** *R*-NPB, **h** *S*-NPB, and **i** racemic-NPB. The axial Br atoms and associated interactions are omitted for clarity. The out-of-plane distortions of equatorial Br atoms in **d** and **e** are clearly seen in **g** and **h** for *R*-NPB and *S*-NPB, respectively. Green, red/purple, black, blue, and pink spheres denote Pb, Br, C, N, and H atoms, respectively.

Single crystals of *R*/*S*/racemic-NPB (NPB = 1-(1-naphthyl)ethylammonium lead bromide) were grown by slowly cooling an aqueous HBr solution of stoichiometric amounts of PbBr_2_ and R/S/racemic-NEA (NEA = 1-(1-naphthyl)ethylamine) (see “Methods” section). All three compounds crystallize as 2D HOIPs, with anionic [PbBr_4_]^2−^ layers of corner-sharing PbBr_6_ octahedra separated by bilayers of NEA^+^ spacer cations (Fig. 1a–c). Racemic-NPB crystallizes in the centrosymmetric *P*2_1_/*c* space group, whereas *R*- and *S*-NPB adopt the chiral *P*2_1_ space group (see Supplementary Table 1 for crystallographic data). In all three compounds, any set of four interconnected PbBr_6_ octahedra forms a puckered square pattern due to the tilting of adjacent octahedra (Fig. 1d–f), causing equatorial Pb–Br–Pb bond angles to deviate significantly from the ideal 180° for an undistorted perovskite sheet. Whereas racemic-NPB features symmetric tilting distortions, resulting in a single Pb–Br–Pb angle of 152° (Fig. 1f), both *R*- and *S*-NPB exhibit significant tilting asymmetry with two widely disparate equatorial Pb–Br–Pb angles of 143° and 157°, corresponding to two distinct equatorial Br atoms (denoted with purple and red spheres, respectively, in Fig. 1d, e).

The equatorial bond angle disparity in chiral NPB likely follows from asymmetric hydrogen (H)-bonding associated with the in-plane Br atoms of the inorganic [PbBr_4_]^2−^ layers (for H-bonding distances and angles, see Supplementary Table 2). As can be seen in Supplementary Fig. 1, each axial (i.e., out-of-plane) Br atom forms the same number of H-bonds in both chiral- and racemic-NPB, i.e., two H-bonds with nearest H-atoms of NH_3_^+^ groups in the adjacent organic layer, as commonly found among 2D HOIPs. Thus, in all three compounds (racemic and chiral), H-bonding to axial Br atoms is symmetric for opposite sides of the same inorganic layer. In contrast, for the chiral NPB, when considering the two distinct equatorial (i.e., in-plane) Br atoms within a given inorganic layer (Fig. 1g, h), each purple-labeled Br atom H-bonds with both NH_3_^+^ (H_2_N-H…Br = 2.92 Å) and α-CH_3_ (H_2_C-H…Br = 2.83 Å) groups of the NEA^+^ spacer on one side of the inorganic layer, but with only the NH_3_^+^ (H_2_N-H…Br = 2.87 Å) group of the NEA^+^ spacer on the opposite side of the inorganic layer. For the red-labeled Br atoms, the corresponding H…Br contacts exceed the van der Waals limit of 3.05 Å, suggesting substantially reduced H-bonding interactions. The equatorial Br atoms thus incur out-of-plane distortions that propagate helically about the 2_1_-screw axis parallel to the *b*-axis (Fig. 1d, e); the spirals formed by the bonds that connect the two types of Br via adjoining Pb atoms (also indicated in Fig. 1d, e) exhibit opposite yet dissimilar helicities, and the ensuing net crystallographic helicity of Br distortions in *R*-NPB (Fig. 1d) is opposite to that in *S*-NPB (Fig. 1e). In the racemic-NPB, this type of helical distortion does not occur. Here, each of the equatorial Br atoms H-bonds to a single NH_3_^+^ (H_2_N-H…Br = 2.59 Å) group on either side of the inorganic layer, with all the equatorial Br atoms being coplanar (Fig. 1f, i). The peculiar H-bonding found for the equatorial Br atoms in chiral NPB derives from the specific organic tethering group configuration. On either side of the inorganic layer, terminal -CH-NH_3_^+^ bonds of chiral NEA^+^ cations are oriented nearly parallel with respect to one another and slightly offset across the inorganic layer (Fig. 1d, e). In racemic-NPB, the -CH-NH_3_^+^ bonds orient in a more typical crisscross fashion (Fig. 1f), leading to a hydrogen-bonding pattern frequently observed among 2D HOIPs.

The structural symmetry determined using PLATON’s^24^ ADDSYM tool yields the same *P*2_1_ chiral space group for mutually isolated organic and inorganic frameworks in *R*- and *S*-NPB (Supplementary Fig. 2). The chiral NEA^+^ spacers assemble into a *P*2_1_ sublattice and transfer the *P*2_1_ chiral symmetry to the inorganic sublattice (Pb and Br atoms in polar *C*_1_ point group) by inducing in-plane tilting asymmetry and out-of-plane helical 2_1_ screw distortions, as discussed above. Wilson statistics and cumulative intensity distributions of X-ray reflections both confirm the noncentrosymmetric inorganic layers in chiral NPB (Supplementary Figs. 3 and 4). In contrast, the [PbBr_4_]^2−^ framework in the racemic-NPB is centrosymmetric (*P*2_1_/*c*), with Pb and Br atoms in the *C*_i_ point group. Bond length distortions for *R*-, *S*-, and racemic-NPB, ${\mathrm{{\Delta} }}d = \left( {\frac{1}{6}} \right){\sum} {\left( {d_i - d} \right)^2} /d^2$ (where $d_i$ denotes the six Pb–Br bond lengths and $d$ is the mean Pb–Br bond length), as well as the bond angle variances, $\sigma ^2 = \mathop {\sum}\nolimits_{i = 1}^{12} {\left( {\theta _i - 90} \right)^2} /11$ (where $\theta _i$ denotes the individual *cis* Br–Pb–Br bond angles), quantify the distortions of individual PbBr_6_ octahedra relative to an undistorted octahedron (Supplementary Table 3). Both ${\mathrm{{\Delta} }}d$ and $\sigma ^2$ values for chiral *R*- and *S*-NPB substantially exceed those for racemic-NPB (Supplementary Table 3) and are among the largest values reported for the <100>-oriented 2D lead bromide HOIP class (Supplementary Fig. 5). Such substantial symmetry-breaking distortions in the inorganic framework, owing to the chirality transfer from the chiral NEA^+^ spacer cations, are crucial for determining the associated electronic structure and lead to an emergent Rashba–Dresselhaus spin-splitting of electronic bands (discussed below) in chiral *R*- and *S*-NPB, otherwise absent when employing a racemic mixture of NEA^+^ spacers.

Critically, individual symmetries of organic and inorganic sublattices can differ, although the use of chiral spacer cations generally entails a global chiral crystallographic description of the HOIP. We underscore the above point by comparing to the previously reported 2D lead iodide HOIP comprising chiral cations, i.e., *R*/*S*-1-methyl benzylammonium lead iodide (MBPI)^18,25^. Temperature-dependent (298, 200, and 100 K) single-crystal X-ray diffraction for *S*-MBPI as an example indicates a nominally centrosymmetric *Pnma* (i.e., *P* 2_1_/*n* 2_1_/*m* 2_1_/*a*) space group for the inorganic framework despite an overall chiral *P*2_1_2_1_2_1_ description for the hybrid. Note that the inorganic framework scatters X-rays more strongly than the organic component, and the positions of Pb and I atoms, therefore, predominantly dictate the space group determination by crystallographic indexing based on Wilson statistics; a <*E*^2^ − 1> value close to 1 is obtained from Wilson statistics, suggesting a centrosymmetric space group (Supplementary Fig. 3). The cumulative intensity distribution plot also points to a centrosymmetric space group (Supplementary Fig. 4). However, structure refinement in the *Pnma* space group is precluded by the chiral organic cations, thereby requiring an alternative *P*2_1_2_1_2_1_ chiral space group, as inferred from the observed total systematic absences. The global structure of *S*-MBPI could be successfully refined in the *P*2_1_2_1_2_1_ chiral space group at all three temperatures investigated (Supplementary Table 4), in agreement with an earlier report on its 298 K structure^25^. However, despite the overall chiral space group, our post-refinement symmetry analysis using PLATON suggests a *Pnma* space group for the isolated [PbI_4_]^2−^ framework (i.e., excluding organic cations) (Supplementary Fig. 2). In contrast to *S*-NPB and *R*-NPB, the observations for S-MBPI clearly point to a predominantly centrosymmetric inorganic [PbI_4_]^2−^ framework to within the default positional/angular tolerance criteria used in the PLATON analysis.

Importantly, S-MBPI lacks the asymmetric H-bonding interactions or helical distortions found in chiral NPB and exhibits nearly flat perovskite layers, similar to racemic-NPB (Supplementary Fig. 6). The in-plane tilting distortion is relatively smaller, with equatorial Pb–Br–Pb angles of 151° and 157°, and the computed *σ*^2^ value is about half the value found in chiral NPB (Supplementary Table 3). We ascribe the organic-to-inorganic structural chirality transfer in NPB and a lack of detectable transfer in MBPI, at least in part, to the relative strengths of associated H-bonding interactions [e.g., ∆*H*_(H…Br)_ > ∆*H*_(H…I)_] that mainly determine the templating influence of chiral organic spacers. Our attempts to grow single crystals of an iodide analog of NPB led to a 1D hybrid comprising a face-sharing [Pb_2_I_6_]^2−^ framework (Supplementary Fig. 7 and Supplementary Table 5). On the other hand, the chiral lead bromide analog of MBPI readily crystallizes into a similar 1D [Pb_2_Br_6_]^2−^ hybrid^25^, although the target 2D phase could be stabilized in thin films^17^. While we cannot directly compare bromide/iodide analogs for the same chiral organic cation (from a single-crystal structure standpoint), the key point is that detectable organic-to-inorganic structural chirality transfer does not inevitably follow from the use of a chiral cation within the 2D HOIPs, but additionally relies on the detailed H-bonding interactions that couple the organic and inorganic sublattices. Furthermore, as seen below, the degree of structural chirality transfer (and associated structural distortions in the lead halide framework) is found to have a significant impact on the electronic structure of the resulting HOIP.

### Circular dichroism and photoluminescence

**Fig. 2:**
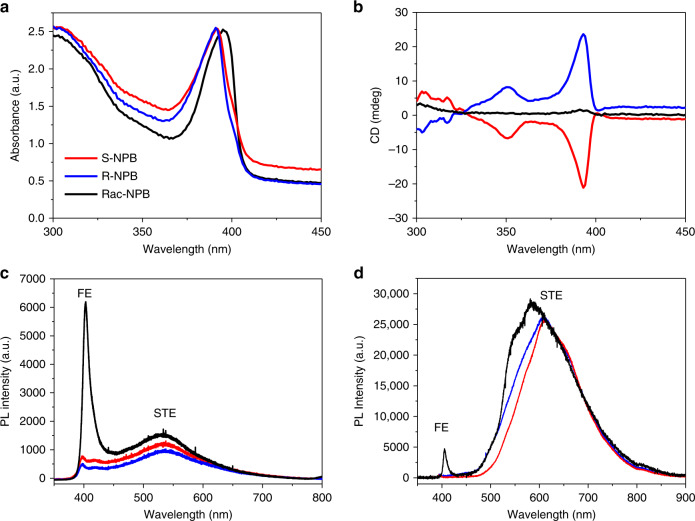
Optical properties of chiral- and racemic-NPB. **a** UV–Vis linear absorption spectra and **b** circular dichroism (CD) spectra recorded at room-temperature for thin films of racemic-, *R*- and *S*-NPB. **c**, **d** Photoluminescence spectra from single crystals of racemic-, *R*- and *S*-NPB obtained at **c** room-temperature using a 325 nm laser source, and at **d** 7 K using a 266 nm laser source; FE and STE denote free excitons and self-trapped excitons, respectively.

Thin films of *R*-, *S*- and racemic-NPB were deposited by spin-coating DMF solutions of corresponding single crystals (see “Methods” section and Supplementary Fig. 8). The linear absorption spectra for *R*-, *S*- and racemic-NPB films all reveal a sharp resonance centered at ~390 nm that corresponds to excitons confined within the 2D lead bromide layers (Fig. 2a). The excitonic bands in *R*- and *S*-NPB are blue-shifted by ~30 meV relative to racemic-NPB, due to more pronounced structural distortions in the former^26^. Circular dichroism (CD) measures differential absorption of left and right circularly polarized light passing through a chiral system and can establish the absolute configuration (i.e., handedness) of chiral enantiomers. Quantum theory relates the CD intensity in a chiral molecule to the imaginary part (Im) of the dot product of electric ($\vec \mu _{12}$) and magnetic ($\vec m_{21}$) dipole transition moments^20^:

1 $${\mathrm{CD}}_{{\mathrm{molecule}}} \propto {\mathrm{Im}}\left[ {\vec \mu _{12}.\vec m_{21}} \right],$$

where the indices 1 and 2 denote, respectively, the initial and final chiral molecule quantum states. For a non-zero CD signal, $\vec \mu _{12}$ and $\vec m_{21}$ cannot be orthogonal, a condition satisfied only by chiral point groups devoid of inversion and mirror symmetries. The CD spectra from *R*- and *S*-NPB thin films (Fig. 2b) change signs due to their opposite absolute configurations but are otherwise identical within the experimental error, while racemic-NPB, as expected, shows a negligible CD signal. The distinct CD bands centered at 393 nm for *R*- and *S*-NPB correspond to the excitonic absorption in Fig. 2a. The linear absorption spectrum at 50 K for the chiral NPB reveals a 2D-type band-edge absorption onset at 3.54 eV that appears as a step-like feature preceding the exciton band (Supplementary Fig. 9). Consequently, we assign the higher energy CD band centered at 351 nm for chiral NPB as due to transitions into the continuum (i.e., above bandgap) states. It is noteworthy that the CD spectrum reveals these band-edge-like transitions even at room temperature, while they are not resolved in the room-temperature linear absorption spectrum due to the inhomogeneous broadening of the exciton absorption band (Fig. 2a). The exciton binding energy (*E*_b_) of ~0.38 eV, estimated from the energy difference between excitonic and continuum CD bands, matches the value obtained from the 50 K linear absorption (Supplementary Fig. 9).

**Fig. 3:**
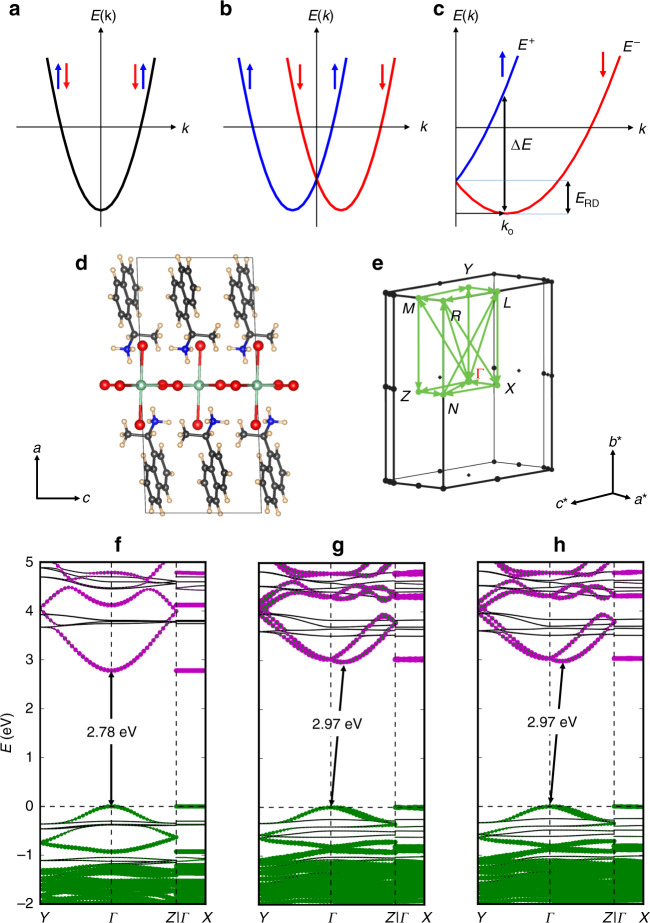
RD spin-splitting in chiral NPB. **a** Schematic representation of a two-fold spin-degenerate electronic band in a conventional semiconductor. **b**, **c** Spin-polarized sub-bands (red: down spin; blue: up spin) separated in k-space due to the SOC and inversion asymmetry along one dimension of an RD semiconductor. $E^ +$ and $E^ -$ denote the inner and outer spin-polarized branches created by the RD spin-splitting. *∆E* denotes the energy difference between the two branches at the characteristic momentum (**k**_0_), and $E_{{\mathrm{RD}}}$ is the characteristic RD energy. **d** A representative unit cell of the relaxed *S*-NPB structure in real space and **e**, its Brillouin zone showing the k-path in reciprocal space used for band plotting in **f**–**h**. For the theoretical structures, cell axes were chosen to be consistent for racemic, *R*- and *S*-NPB such that the stacking direction is always along the *a*-direction, and the inorganic layers are parallel to the *b–c* plane. **f**–**h**, The computed DFT+HSE06 electronic band structures of **f** racemic-NPB, **g** *S*-NPB, and **h** *R*-NPB shown along selected k-paths. The atomic contributions to the electronic continuum bands are identified for Pb (magenta), Br (green), and organic-derived (black) states. The two-fold spin-degenerate lowest conduction band in **f** racemic-NPB splits into upper and lower branches in both **g** *S*-NPB and **h** *R*-NPB mainly along the Γ–*Z* direction of reciprocal space due to inversion asymmetry in the [PbBr_4_]^2−^ perovskite layers of *S*-NPB and *R*-NPB. Full band structures of *R*-, *S*-, and racemic-NPB for all the k-paths are presented in Supplementary Fig. 13.

The opposite CD signals in the inorganic-derived part of the spectra for chiral *R*- and *S*-NPB can, in part, arise from the crystallographic handedness induced by structural chirality transfer. However, *R*- and *S*-MBPI also exhibit inorganic-derived excitonic and interband CD bands despite their nominally centrosymmetric lead iodide layers, as discussed above^18^. Indeed, the frontier orbitals at the conduction and valence band edges derive from the inorganic framework for all the 2D HOIPs studied here (Fig. 3f–h), and no significant direct contribution arises from chiral organic cations to the associated band edge or excitonic transitions. The CD bands in chiral NPB can originate from the coupling of dipole transition moments in the inorganic framework and the chiral organic spacer cations (the chiral screening medium surrounding the inorganic framework), as previously observed in inorganic semiconductor nanocrystals capped with chiral surface ligands^19^. The same dipolar interactions with the chiral, organic screening medium can induce excitonic CD signals in *R*- and *S*-MBPI despite their nominally centrosymmetric inorganic layers^18,27^, thus suggesting that an induced CD does not always imply substantial chirality transfer from organic to inorganic layers.

The room-temperature photoluminescence (PL) spectra measured from single crystals (Fig. 2c) reveal a moderate excitonic emission (centered at 402 nm) for racemic-NPB, which is quenched in the chiral *R*- and *S*-NPB. In addition, all three HOIPs exhibit a weak, spectrally shifted broad PL band in the 450–800 nm range, which significantly increases in intensity at 7 K (Fig. 2d). Such a broad PL band may be attributed to exciton self-trapping within excited state transient lattice distortions (i.e., polaron formation), mediated by strong electron-phonon coupling and defects induced during crystal growth^28^. The PL behavior in chiral- vs. racemic-NPB indeed agrees with a previous empirical correlation—i.e., that the intensity ratio of narrow free-excitonic emission to broad self-trapped excitonic emission in <100>-oriented lead bromide HOIPs decreases with increasing structural distortions in the ground state^29^. Despite the substantial distortions in chiral NPB (Supplementary Fig. 5), the relatively weak room-temperature broad PL emission suggests competing non-radiative decay channels for the self-trapped excitons^30^. The lack of free-excitonic emission in chiral NPB is, in part, ascribed to asymmetry-induced changes in the electronic band structures, as discussed below.

### Rashba–Dresselhaus spin–orbit coupling and conduction band splitting

Spin–orbit coupling (SOC) describes a relativistic effect, relevant especially for heavy atoms. Pb-based HOIPs are gaining substantial interest in spintronics owing to a strong SOC induced by the heavy Pb constituent and structural flexibility provided by the organic cation^31,32^. In Pb-containing HOIPs, the SOC-induced energy splitting of the Pb-6*p*-derived conduction band (CB) is of the order of eV^33–35^. The SOC can be rationalized approximately as the Zeeman-like interaction of electron spin with an effective magnetic field experienced by an electron (in its rest frame) moving in an electric field^7^. In non-magnetic systems, SOC conserves time-reversal symmetry (TRS), i.e., *E*↑(**k**) = *E*↓(−**k**), where *E* is the single-particle (electron or hole) band energy, **k** represents the crystal momentum (Bloch wavevector), and the arrows correspond to states of *s* = +1/2 and −1/2 character. TRS, in combination with inversion symmetry [i.e., *E*↑(**k**) = *E*↑(−**k**)], leads to a two-fold spin degeneracy for the up (↑) and down (↓) spins (Fig. 3a). When the inversion symmetry is broken, a strong SOC lifts this two-fold spin degeneracy. The emergence of the ensuing dispersion relations near the CB minimum along one dimension of an RD system is schematically illustrated in Fig. 3a–c. Without SOC, the CB minimum is located at the Γ-point in this illustration. Upon including SOC, the energy bands about the CB minimum assume the general form^2,32^:

2 $$E^ \pm ({\mathrm{k}}) = \frac{{\hbar ^2{\mathbf{k}}^2}}{{2m}} \pm \lambda |{\mathbf{k}}|,$$

where **k** is the electron wavevector, and $\lambda$ is the Rashba (Dresselhaus) coupling parameter for a pure Rashba (Dresselhaus) SOC effect. $E^ +$ and $E^ -$ are called the spin-polarized sub-branches (Fig. 3b, c). Owing to the k-space separation of electronic bands (Fig. 3b), forward and backward moving carriers have opposite spins at the band extrema (spin-momentum locking). This effect was first noted by Dresselhaus in strained zinc-blende III–V semiconductors^3^, then by Rashba in Wurtzite structures^4^ and later generalized by Bychkov and Rashba for 2D electron gases (2DEG)^2^. In this paper, we use the combined term, RD splitting, throughout since Rashba and Dresselhaus SOC effects can occur in 2D systems either exclusively or simultaneously with characteristic spin textures for each case^32^. A detailed discussion of the RD effect for quasi-2D systems was recently given by Even and coworkers^32^. Opposite spin textures associated with the spin-split sub-bands in opposite momentum directions (Fig. 3b) due to the RD SOC effect lead to circular photogalvanic effects^36^ and enable interconversion between charge and spin currents (by Edelstein and inverse Edelstein effects)^22,23^, of interest for spintronic technologies.

To examine the effect of chirality-induced inversion asymmetry on the electronic structures of chiral *R*- and *S*-NPB, we have computed the associated electronic band structures using DFT-based first-principles calculations with the all-electron electronic structure code FHI-aims^37^. The code is a high-precision implementation of current density-functional methods^38,39^, including an implementation of SOC that has been successfully benchmarked^40^ and used to calculate band structures of complex HOIPs in previous works^35,41–43^ (see “Methods” section for more details). The input structures for the 2D HOIPs were obtained from our single-crystal X-ray experiments. Geometry optimizations were conducted using dispersion-corrected semilocal DFT (PBE^44^ generalized-gradient approximation including the Tkatchenko–Scheffler (TS) van der Waals correction^45^, PBE+TS for short); computationally relaxed lattice parameters fall within 2.5% of the experimental values, while associated bond angles fall within 7° (Supplementary Table 6 and Supplementary Figs. 10–12). Figure 3f–h shows the electronic band structures of racemic-, *R*- and *S*-NPB calculated with DFT-HSE06+SOC and decomposed into Pb- (purple), Br- (green) and organic-derived (black) states. For all three structures, the frontier orbitals at the band edges derive from the inorganic sublattice so that a type Ib quantum well results^35^. In agreement with other Pb-based hybrid perovskites^46^, the conduction band maximum (CBM) and valence band minimum (VBM) consist mainly of Pb- and halogen-derived states, respectively. The calculated bandgaps are the same for *R*- and *S*-NPB (2.97 eV) and slightly higher than the predicted bandgap of 2.78 eV for racemic-NPB.

In line with the RD picture, chiral *R*- and *S*-NPB exhibit characteristic splitting of an otherwise two-fold degenerate conduction band (CB) away from the Γ-point (Fig. 3g, h), whereas the centrosymmetric racemic-NPB does not show CB splitting (Fig. 3f). Broken inversion symmetry in the chiral lead bromide layers results in CB splitting into upper and lower ﻿spin-polarized branches along the Γ–*Z* path. This k-path coincides with the in-plane [100] crystallographic direction in the 2D [PbBr_4_]^2−^ layers (see Fig. 1d, e), along which the local geometry fluctuates as the equatorial Pb–Br–Pb bond angles alternate between 143° and 157° (Fig. 1d, e). The lack of band dispersion along the Γ-X path (i.e., coinciding with the out-of-plane [001] layer-stacking direction, which is perpendicular to the 2D inorganic plane in Fig. 1a, b) is in accord with the confinement and localization of inorganic-derived states in the out-of-plane direction. The Γ–Y path coincides with the in-plane [010] crystallographic direction (Fig. 1d, e) along which the same kind of Br atoms— i.e., either purple- or red-type— and hence the associated Pb–Br–Pb bond angles propagate by 2_1_-screw translational symmetry (Fig. 1d, e). So, the *C*_2_ rotational axis for chiral NPB (corresponding to *P*2_1_ space group) points along the Γ–Y path, thereby explaining the minimal spin-splitting along this direction. This configuration contrasts with conventional 2D Rashba systems such as inorganic quantum well (QW) heterostructures^32,47^ or BiTeI^48^, which exhibit the *C*_*n*V_ symmetry and where the *C*_*n*_ (*n* = 2, 3) axis coincides with the stacking direction. Therefore, as suggested in earlier work on quasi-2D systems by Even and coworkers^32^, the situation in chiral NPB corresponds to a 1D problem with dominant SOC contributions only along the [100] crystallographic direction (Fig. 1d, e).

We describe an effective RD coupling parameter, $\lambda _{{\mathrm{RD}}} = \frac{{{\Delta} E}}{{2{\mathbf{k}}_0}}$, where ${\mathbf{k}}_0$ denotes the momentum-offset from the Γ-point and ${\Delta} E$ is the energy difference between the upper ($E^ +$) and lower ($E^ -$) conduction band branches at ${\mathbf{k}}_0$. $E_{{\mathrm{RD}}}$ is the characteristic energy difference between ${\mathbf{k}} = {\mathbf{k}}_0$ (i.e., the bottom of the parabola) and ${\mathbf{k}} = 0$ (i.e., Dirac point where the two parabolas intersect), which for the exact parabolic dispersion, would amount to one-fourth of ${\Delta} E$ (Fig. 3c)^32^. Based on our DFT-HSE06+SOC band structure calculations, ${\mathrm{{\Delta} }}E^{{\mathrm{{\Gamma} }} - {\mathrm{Y}}} = 0.01$ eV and ${\mathrm{{\Delta} }}E^{{\mathrm{{\Gamma} }} - {\mathrm{Z}}} = 0.22$ eV, with corresponding RD coupling parameters of $\lambda _{{\mathrm{RD}}}^{{\mathrm{{\Gamma} }} - {\mathrm{Y}}} = 0.28$ eV·Å and $\lambda _{{\mathrm{RD}}}^{{\mathrm{{\Gamma} }} - {\mathrm{Z}}} = 1.52$ eV·Å for CBs in both *R*- and *S*-NPB along the Γ–*Y* and Γ–*Z* paths, respectively. The predicted $\lambda _{{\mathrm{RD}}}^{{\mathrm{{\Gamma} }} - {\mathrm{Z}}}$ of 1.52 eV·Å and estimated $E_{{\mathrm{RD}}}$ ($\sim \!{\Delta} E/4$)^32,49^ of 55 meV in chiral NPB are orders of magnitude higher than those found in QW heterostructures such as InAlAs/InGaAs ($\lambda \approx 0.07$ eV·Å; $E_{{\mathrm{RD}}} \approx 1$ meV)^50^. These values are indeed similar to those recently observed in the 2D phenethylammonium lead iodide HOIP using optical spectroscopies^36,49^, although the precise origin of the bulk inversion asymmetry is still unclear given its centrosymmetric X-ray crystal structure with a symmetrical disposition of in-plane bond angles and relatively low distortions (in contrast to chiral NPB)^26^. We speculate that an instantaneous band splitting in phenethylammonium lead iodide can arise from dynamic structural changes induced by temperature and/or photoexcited coherent phonon modes, as recently proposed for methylammonium lead iodide 3D perovskite^51,52^. Nonetheless, in contrast with the substantially lower RD splitting predicted for noncentrosymmetric lead iodide-based HOIPs ($E_{{\mathrm{RD}}} \ll 10\,{\mathrm{meV}}$)^53^, we ascribe the relatively large in-plane RD CB spin-splitting in the chiral NPB compounds to a considerable asymmetry in the in-plane PbBr_6_ octahedral tilting distortions—i.e., widely disparate Pb–Br–Pb bond angles (Fig. 1d, e)—likely inducing large electric fields and thus large RD SOC, in agreement with our simulations of model structures with distorted vs. undistorted inorganic layers (Supplementary Fig. 14). Furthermore, the opposite chirality in the inorganic layers leads to opposite spin textures for the spin sub-bands between *R*-NPB and *S*-NPB, thus enabling a unique control of RD spin polarization using the chiral organic cations (Supplementary Fig. 15).

In *R*- and *S*-NPB, the resulting CB minima due to RD splitting are offset in k-space from the VB maxima (indicated by black arrows in Fig. 3g, h), resulting in a momentum-forbidden indirect bandgap close to Γ-point. In contrast, the bandgap in racemic-NPB is direct and momentum-allowed (Fig. 3f). We thus speculate that the quenched free-excitonic PL emission in the chiral NPB compounds may arise in part due to the indirect transition caused by substantial RD CB splitting^52,54^. The frontier VB in NPB systems is primarily comprised of Br-derived states (Fig. 3f–h) and, owing to the low (compared to Pb) SOC effects associated with the lighter Br, RD VB spin-splitting is expectedly small in chiral NPB (Fig. 3g, h)^34^. For comparison, we have also computed the DFT-HSE06+SOC electronic band structure of chiral *S*-MBPI (Supplementary Fig. 16), which, however, shows much lower CB splitting of ${\mathrm{{\Delta} }}E^{{\mathrm{{\Gamma} }} - {\mathrm{Z}}}$ =0.025 eV due to the previously discussed nearly centrosymmetric [PbI_4_]^2−^ layers, thus reflecting the essential prerequisite of substantial local symmetry-breaking for a large RD spin-splitting.

## Discussion

2D HOIPs exhibiting quantum and dielectric confinement of carriers are potential RD candidates^49^, in analogy with the inorganic 2DEGs, provided the perovskite layers lack inversion symmetry. Most of the known 2D HOIPs, however, adopt centrosymmetric crystal structures, lacking the bulk and/or site inversion asymmetries that are prerequisite to the RD SOC effect (neglecting dynamical effects)^32,55^. Our present work shows that the use of enantiopure chiral building blocks provides a pathway to induce inversion asymmetry in otherwise centrosymmetric HOIPs, reflecting an additional degree of freedom in HOIP design. Notably, the surrounding chiral cations, the presence of which induces a chiroptical response, need not always entail a significant structural chirality within the inorganic layers; chirality can be structurally transferred to the inorganic layers primarily when mediated by suitably strong hydrogen-bonding interactions with chiral cations, as exemplified by the present chiral NPB systems. Moreover, while specific achiral organic cations might also induce symmetry-breaking distortions in the inorganic layers, the ability to control the associated handedness of these distortions (and associated spin texture in the electronic bands) is made possible by employing chiral organic cations. These structural insights, coupled with theoretical findings in our work, enable the discovery and design of an emerging class of multifunctional hybrid systems with an amalgam of semiconducting, chiroptical, and spin-dependent properties. Indeed, the combination of chiral induced spin selectivity of chiral organic molecules^56–62^ and an intrinsic RD spin-splitting in the inorganic framework is a hitherto unexplored aspect unique to 2D chiral HOIPs and might have practical implications in future HOIP-based spintronics. Further, the structural chirality transfer and ensuing lowering of symmetry within the inorganic sublattice opens up a gateway to other emergent properties such as nonlinear light-matter interactions and piezo-/ferroelectricity, not just in 2D HOIPs but also in related diverse low-dimensional metal halide hybrids.

## Methods

### Materials

(*S*)-(−)-1-(1-naphthyl)ethylamine (≥99%), (R)-(+)-1-(1-naphthyl)ethylamine (≥99%), 1-(1-naphthyl)ethylamine (98%), (*S*)-(−)-α-methyl benzylamine (98%), hydrobromic acid (48 wt.% in H_2_O, ≥ 99.99%), and hydroiodic acid (57 wt. % in H_2_O, distilled, stabilized, 99.95%) were purchased from Sigma Aldrich and used without further purification.

### Synthesis

For growing single crystals of chiral *R*- or *S*-NPB, stoichiometric amounts of PbBr_2_ (45 mg, 0.12 mmol), and *R*- or *S*-1-(1-naphthyl)ethylamine (39 µL, 0.24 mmol) were first dissolved in a mixture of 0.5 ml aq. HBr and 1.2 mL deionized water in a sealed vial with an N_2_ atmosphere at 95 °C. The hot solution was slowly cooled to room temperature over 48 hr. Single crystals of racemic-NPB were grown in a similar way from a solution of racemic 1-(1-naphthyl)ethylamine (39 µL, 0.24 mmol) and PbBr_2_ (45 mg, 0.12 mmol) in 0.5 mL of aq. HBr and 1.2 mL methanol. The as-obtained colorless plate-like crystals were filtered, washed with diethyl ether, and vacuum-dried. Single crystals of 1D *S*-NEA_2_Pb_2_I_6_ (*S*-NPI) were obtained by cooling a hot aq. HI solution of *S*-1-(1-naphthyl)ethylamine (0.25 mmol) and PbI_2_ (0.125 mmol) from 90 °C to room-temperature in 48 h. The as-obtained pale-yellow, needle-like crystals were filtered, washed with diethyl ether, and vacuum-dried. Single crystals of *S*-MBPI were grown by slowly evaporating a solution of (*S*)-(−)-α-methyl benzylamine (25 µL, 0.2 mmol) and PbI_2_ (45 mg, 0.1 mmol) in 1 mL aq. HI and 1 mL methanol at room temperature under N_2_ atmosphere. The as-obtained orange-red, needle-like crystals were filtered, washed with copious amounts of diethyl ether and vacuum-dried. To fabricate thin films, 0.2 M solutions of R/S/racemic-NPB single crystals in DMF were spin-cast on glass substrates (precleaned by ultrasonication in IPA for 10 min followed by Ar-O_2_ plasma treatment for 10 min) at a spin speed of 3000 rpm for 30 s and annealed at 120 °C for 10 min in an N_2_ glove box. Similarly, thin films of *S*-MBPI were spin-coated from 0.2 M DMF solution and annealed at 100 °C for 5 min in an N_2_ glove box.

### Characterization

Single-crystal X-ray diffraction (XRD) was performed at 298 K on a Rigaku XtaLAB Synergy-S diffractometer (Mo-K$\alpha$ radiation, *λ* = 0.710 Å; X-ray tube operating at 50 kV and 30 mA) for the chiral *R*- and *S*-NPB and *S*-MBPI single crystals and on a Bruker APEX II CCD diffractometer (Mo-K$\alpha$ radiation, *λ* = 0.710 Å; X-ray tube operating at 50 kV and 30 mA) for the racemic-NPB and *S*-NPI. For *S*-MBPI, X-ray diffraction data were collected subsequently at 298, 200, and 100 K using an Oxford Cryosystem for temperature control. Structure solutions were obtained by SHELXS direct methods and refined using SHELXL least-squares method within the Olex^2^ crystallographic package. A post-refinement analysis for missing symmetry has been carried out for both full structures and isolated inorganic frameworks (i.e., after manually deleting the organic component) using the ADDSYM tool implemented in the PLATON program. Powder XRD was carried out for thin films using a PANalytical Empyrean powder X-ray diffractometer (CuKα radiation) operating at 45 kV and 40 mA. Room-temperature circular dichroism (CD) spectra were measured for thin films (on glass substrates) of R/S/racemic-NPB using an AVIV 420 CD spectrophotometer with 1 nm s^−1^ scan speed. UV–Vis absorption spectra for thin films were obtained at room temperature using a Shimadzu UV-3600 UV–Vis-NIR spectrophotometer. For low-temperature absorption measurements, thin films of *R*-, *S*- and racemic-NPB deposited on sapphire substrates were transferred into a He Cryostat and cooled down to low temperatures using a closed-cycle refrigerator. An incandescent light source from a Xenon lamp dispersed through a monochromator was focused on the sample and detected by an ultraviolet-enhanced silicon photodetector. Transmission spectra were measured using a lock-in amplifier, and optical densities were subsequently calculated. The PL emission from single crystals was recorded at room temperature on a Horiba Jobin Yvon LabRam ARAMIS spectrophotometer using a HeCd (325 nm) laser as an excitation source, 1800 gr/min diffraction grating, and an InGaAs detector. For 7 K PL measurements on single crystals, a solid-state laser operating at 266 nm was used as the pump excitation with an oblique incidence angle of 45°, and the PL emission was collected in reflection geometry and measured with a fiber spectrometer (Ocean Optics USB4000). Thin crystals of NPB were cooled down in a cryostat with optical windows (Cryocooler Model SRDK-205).

### First-principles calculations

The all-electron electronic structure code FHI-aims^37^ was employed to perform the first-principles density-functional theory (DFT) calculations. All calculations are based on numeric atom-centered orbital (NAO) basis sets. The massively parallel simulations were assisted by the ELSI infrastructure^63,64^ and ELPA eigenvalue solver^65^. Full relaxation of lattice parameters and atomic coordinates for all systems was performed with the semilocal Perdew–Burke–Ernzerhof (PBE) functional^44^ plus the Tkatchenko–Scheffler (TS) pairwise dispersion scheme for van der Waals (vdW) interactions^45^. FHI-aims “tight” numerical defaults were used, and the k-point grid was set to $(2 \times 4 \times 4)$. In the experimental structures, the layer-stacking direction is along the *c*-axis for *R*- and *S*-NPB while it is along *a*-axis for the racemic-NPB (Supplementary Table 1). For the calculations, we chose consistent labeling of crystal axes for all the structures so that the stacking direction is always along *a*-axis while the *b*- and *c*-axes (*c* > *b*) span the plane of inorganic layers (see Fig. 3d, e). Hence, the short dimension of the k-space grid corresponds to the long, out-of-plane lattice direction in real space. Regarding energy band structures, it is well-known that DFT-GGA suffers from the electronic delocalization error^66^ that can lead to too small bandgaps or wrong ordering of electronic levels^67^. As the lowest-energy crystal structure and electronic structure are different observables, we here used spin–orbit coupled hybrid DFT on top of the PBE+TS relaxed atomic structures for the description of electronic properties, offering a good compromise between affordability in terms of computational cost and known accuracy for HOIPs, as we have shown in past work^35,41–43,68^, compared to more computationally involved *GW*^69^. We employed the Heyd–Scuseria–Ernzerhof (HSE06) hybrid density-functional^70,71^ (with 25% Hartree–Fock exchange and a screening parameter of 0.11 bohr^−1^) plus second-variation non-self-consistent spin–orbit coupling (SOC)^40^ within FHI-aims using “intermediate” numerical settings and again a $(2 \times 4 \times 4)$ k-point grid to predict the character of frontier orbitals and band structure properties.

### Spin texture implementation and validation

Spin texture based on SOC calculations can help understand the spin-polarization behavior of individual bands. Spin texture can be defined as the expectation value of the vector of Pauli matrices $\sigma _i$ and this functionality has here been implemented in the FHI-aims code^37^:

3 $$\sigma _{i,n{\mathbf{k}}} = \left \langle {{\Psi} _{n{\mathbf{k}}}} \right|\sigma _i\left| {{\Psi} _{n{\mathbf{k}}}} \right \rangle,\,i = x,y,z.$$

Here, ${\Psi} _{n{\mathbf{k}}}$ is the *n*th crystal orbital associated with the crystal momentum **k**. It is a two-component spinor, which can be expanded using *N* spatial Bloch basis functions $\varphi _{\mu {\mathbf{k}}}$:

4 $${\Psi} _{n{\mathbf{k}}} = \mathop {\sum }\limits_{\mu = 1}^N \left( {C_{\mu n}^\alpha \varphi _{\mu {\mathbf{k}}}^\alpha + C_{\mu n}^\beta \varphi _{\mu {\mathbf{k}}}^\beta } \right).$$

The quantities *C* are the expansion coefficients and the two spinor components associated with each basis function are denoted as *α* and *β*. $\varphi _{\mu {\mathbf{k}}}^\alpha$ and $\varphi _{\mu {\mathbf{k}}}^\beta$ are expressed in vector form as follows.

5 $$\varphi _{\mu {\mathbf{k}}}^\alpha = \left( {\begin{array}{*{20}{c}} {\varphi _{\mu {\mathbf{k}}}} \\ 0 \end{array}} \right),\,\varphi _{\mu {\mathbf{k}}}^\beta = \left( {\begin{array}{*{20}{c}} 0 \\ {\varphi _{\mu {\mathbf{k}}}} \end{array}} \right).$$

They are Bloch basis functions and, summing over individual unit cells and appropriate phase factors, can be further expressed in terms of localized atom-centered orbital basis functions^37,40^. The final expression for the expectation values of the three Pauli matrices can be obtained using the overlap matrix between basis functions *μ* and $\nu, S_{\mu \nu } = \langle \varphi _{\mu {\mathbf{k}}}{\mathrm{|}}\varphi _{\nu {\mathbf{k}}}\rangle$:

6a $$\begin{array}{l}\langle \sigma _{x,n{\mathbf{k}}}\rangle = \langle \sigma _{x,n{\mathbf{k}}}\rangle _{\alpha \alpha } + \langle \sigma _{x,n{\mathbf{k}}} \rangle _{\beta \beta } + \langle \sigma _{x,n{\mathbf{k}}}\rangle_{\alpha \beta } + \langle \sigma _{x,n{\mathbf{k}}}\rangle_{\beta \alpha } \\ = \mathop {\sum }\limits_{\mu = 1}^N \mathop {\sum }\limits_{\nu = 1}^N C_{\mu n}^{\alpha \ast }C_{\nu n}^\beta S_{\mu \nu } + \mathop {\sum }\limits_{\mu = 1}^N \mathop {\sum }\limits_{\nu = 1}^N C_{\mu n}^{\beta \ast }C_{\nu n}^\alpha S_{\mu \nu },\end{array}$$

in which the $\langle \sigma _{x,n{\mathbf{k}}}\rangle _{\alpha \alpha }$ and $\langle \sigma _{x,n{\mathbf{k}}}\rangle _{\beta \beta }$ terms both vanish. Similarly, we have:

6b $$\langle \sigma _{y,n{\mathbf{k}}} \rangle = - i\mathop {\sum }\limits_{\mu = 1}^N \mathop {\sum }\limits_{\nu = 1}^N C_{\mu n}^{\alpha \ast }C_{\nu n}^\beta S_{\mu \nu } + i\mathop {\sum }\limits_{\mu = 1}^N \mathop {\sum }\limits_{\nu = 1}^N C_{\mu n}^{\beta \ast }C_{\nu n}^\alpha S_{\mu \nu },$$

6c $$\langle \sigma _{z,n{\mathbf{k}}} \rangle = \mathop {\sum }\limits_{\mu = 1}^N \mathop {\sum }\limits_{\nu = 1}^N C_{\mu n}^{\alpha \ast }C_{\nu n}^\alpha S_{\mu \nu } - \mathop {\sum }\limits_{\mu = 1}^N \mathop {\sum }\limits_{\nu = 1}^N C_{\mu n}^{\beta \ast }C_{\nu n}^\beta S_{\mu \nu }.$$

We benchmarked the spin texture functionality within our FHI-aims code against results for several reference systems found in the literature, calculated using other codes. The implementation details of SOC vary between different codes, for instance, due to the use of non-self-consistent vs. self-consistent SOC^40^ or due to the use of a pseudopotential vs. all-electron approach. We thus expect a qualitative, but not necessarily an exactly quantitative match to literature results from different codes. In FHI-aims, SOC calculations are performed non-self-consistently^40^, based on an all-electron approach that makes no shape approximation to the potential. An underlying atomic zero-order regular approximation (atomic ZORA) treatment is used to include scalar relativistic effects in the kinetic energy expression, following the specific Eqs. (55) and (56) in ref. ^37^. Three benchmark systems were chosen (Supplementary Figs. 17–19).

For the Au (111) surface, we compared with results published using the OpenMX code^72–74^. In Supplementary Fig. 17a, the bands showing Rashba splitting around the Г point are indexed as the 241st and 242nd bands (corresponding to the 55th and 56th bands calculated by OpenMX)^74^. The calculated spin textures show exactly opposite spin polarizations perpendicular to the momentum **k** in the k_*x*_ – k_*y*_ plane, in qualitative agreement between FHI-aims and OpenMX (Supplementary Fig. 17b, c). The precise locations of the Fermi surfaces in reciprocal space vary somewhat, which we ascribe to differences in SOC and pseudopotential (OpenMX) vs. all-electron (FHI-aims) treatments.

For the 2D hybrid organic–inorganic perovskite (4-BrBzA)_2_PbI_4_^53^, the spin textures for the inner and outer branches of the valence and conduction bands were calculated using FHI-aims and compared with results shown in reference^53^, which were calculated using the VASP code (Supplementary Fig. 18a, b). The spin textures show similar shapes and features.

A third test case, IrBiSe, shows a giant bulk-type Dresselhaus splitting. Along the symmetry line X (0.5, 0, 0) → Г (0, 0, 0), the valence band spin polarization should be parallel to the k-direction^75^. This is confirmed in Supplementary Fig. 19a, b, in which both $\sigma _y$ and $\sigma _z$ vanish, and only $\sigma _x$ shows non-zero values. In contrast, along *X* (0.5, 0, 0) → M (0.5, 0.5, 0), the *z* component of the spin polarization vanishes.

## Supplementary information

Supplementary Information

## Data Availability

Additional data supporting the findings of this work are provided as a Supplementary Information file. Single-crystal structures in this work are available in The Cambridge Crystallographic Data Center (CCDC) database: Racemic-NPB: 2015614 [10.5517/ccdc.csd.cc25ndt2]; S-NPB: 2015618 [10.5517/ccdc.csd.cc25ndy6]; R-NPB: 2015620 [10.5517/ccdc.csd.cc25nf09]; S-MBPI_298 K: 2015617 [10.5517/ccdc.csd.cc25ndx5]; S-MBPI_200 K: 2015616 [10.5517/ccdc.csd.cc25ndw4]; S-MBPI_100 K: 2015619 [10.5517/ccdc.csd.cc25ndz7]; S-NPI: 2015615 [10.5517/ccdc.csd.cc25ndv3]. Single-crystal data are also available in the HybriD^3^ material database and can be accessed using the search fields: “racemic-NEA2PbBr4” (Racemic-NPB), “S-NEA2PbBr4” (S-NPB), “R-NEA2PbBr4” (R-NPB), “S-MBA2PbI4” (S-MBPI) and “S-NEA2Pb2I6” (S-NPI). Relaxed geometries and computed band structures can also be accessed from the HybriD^3^ materials database: “racemic-NEA2PbBr4 [https://materials.hybrid3.duke.edu/materials/dataset/1634]”, “S-NEA2PbBr4 [https://materials.hybrid3.duke.edu/materials/dataset/1630]”, “R-NEA2PbBr4 [https://materials.hybrid3.duke.edu/materials/dataset/1632]”, “S-MBA2PbI4 [https://materials.hybrid3.duke.edu/materials/dataset/1636]”. The whole computational data set is also deposited in the NOMAD repository. Other relevant data can be obtained from the corresponding author or the first author upon reasonable request.
