# Supplementary material for: Organic-to-inorganic structural chirality transfer in a 2D hybrid perovskite and impact on Rashba-Dresselhaus spin-orbit coupling
Source: Nat Commun. 2020 Sep 17;11:4699. doi: 10.1038/s41467-020-18485-7 (PMC7499302; doi:10.1038/s41467-020-18485-7)
Supplement: Supplementary file 1 — Supplementary Information [file 41467_2020_18485_MOESM1_ESM.pdf]

Supplementary Information

Organic-to-inorganic structural chirality transfer in a 2D hybrid perovskite and impact on Rashba-Dresselhaus spin-orbit coupling

Jana *et. al.*

Supplementary Figures

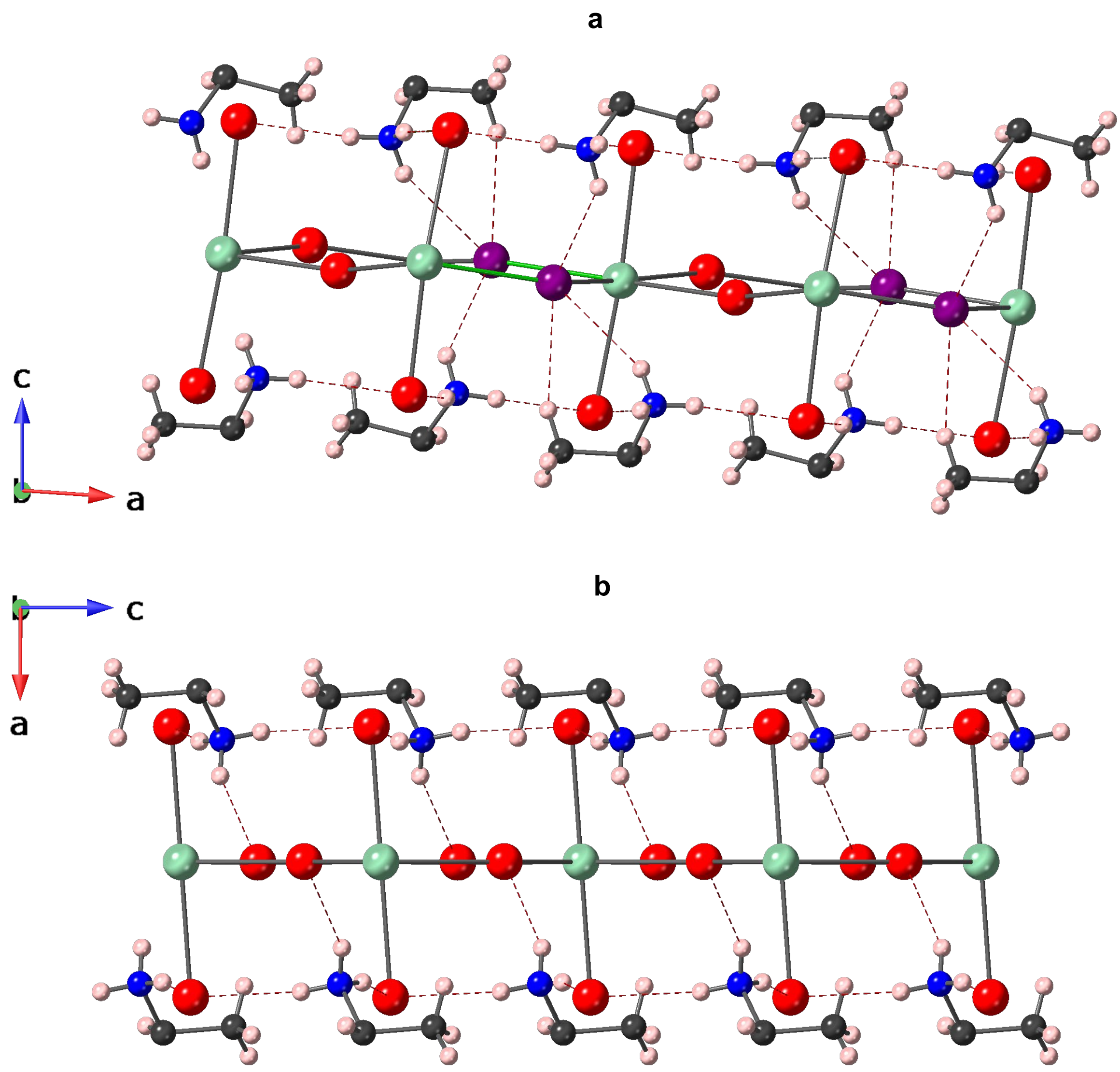

Supplementary Figure 1 | a,b, Hydrogen bonding interactions of axial and equatorial Br atoms with NEA^+ spacer cations in (**a**) R-NPB and (**b**) racemic-NPB. The dotted lines indicate the shortest H---Br contacts. Green, red/purple, black, blue and pink spheres denote Pb, Br, C, N and H atoms, respectively. See **Supplementary Table 2** for hydrogen bond distances and the main text for detailed discussion. Note that the hydrogen bonding interactions in S-NPB (not shown here) are analogous to those in R-NPB.

a S-NPB

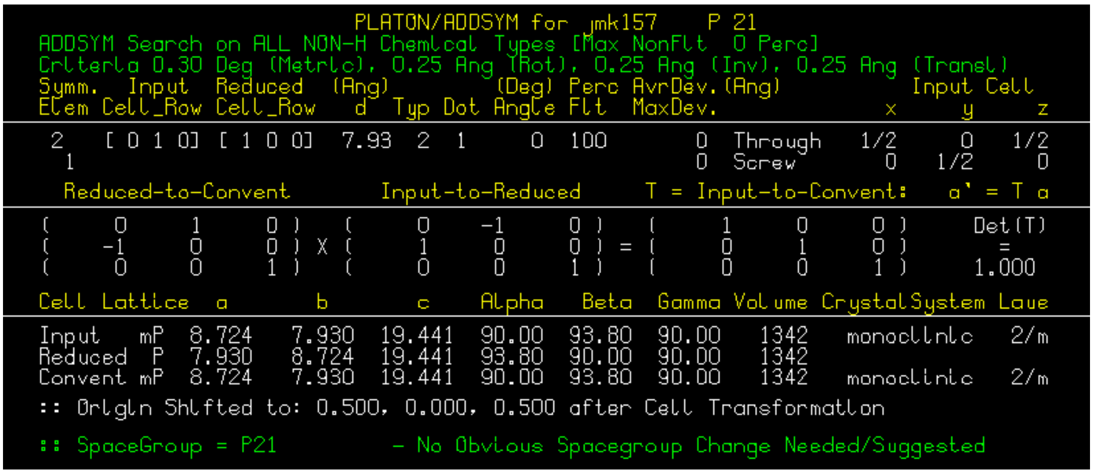

b S-MBPI

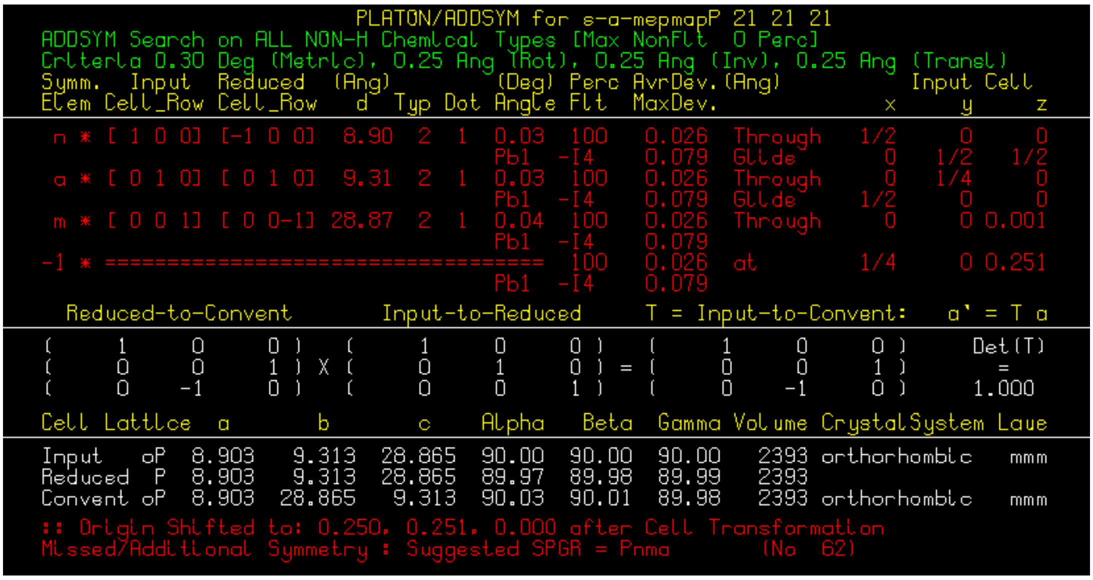

Supplementary Figure 2 | a,b, Post-refinement analysis for missing symmetry using PLATON's ADDSYM tool on isolated inorganic frameworks (i.e., without organic cations) in S-NPB (**a**) and S-MBPI (**b**). All the C, H, and N atoms were manually deleted from the fully refined structure using the CrystalMaker software. The remaining lead halide framework is exported into a CIF file for PLATON symmetry analysis. The analysis includes all Pb and Br/I atoms in the search (i.e., maximum allowed non-fitting atoms = 0 %). Default values of angle criterium (i.e., 0.3° for lattice metrical symmetry) and distance criterium (i.e., 0.25 Å for coinciding atoms for rotational, inversion and translational symmetry elements) were used in the symmetry search. For the lead bromide framework in S-NPB (**a**), there is no change from the original $P2_1$ space group, whereas for the lead iodide framework in S-MBPI (**b**), the PLATON analysis suggests an alternative centrosymmetric *Pnma* space group instead of the input $P2_12_12_1$ (see lowest line in (**b**)).

a

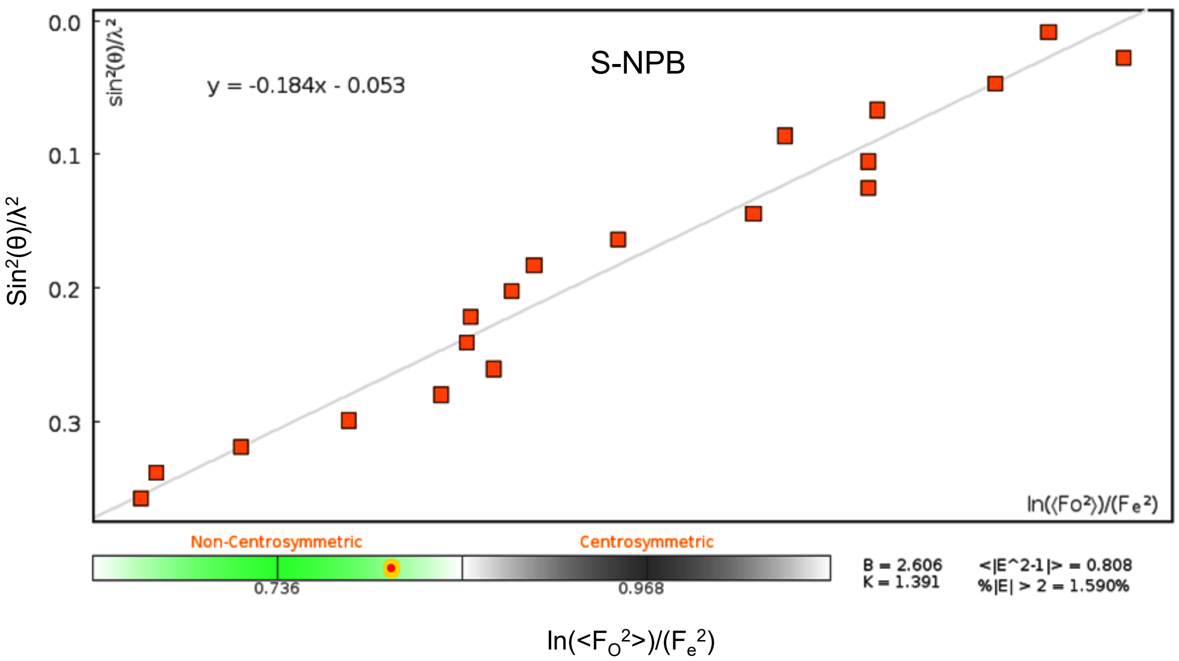

b

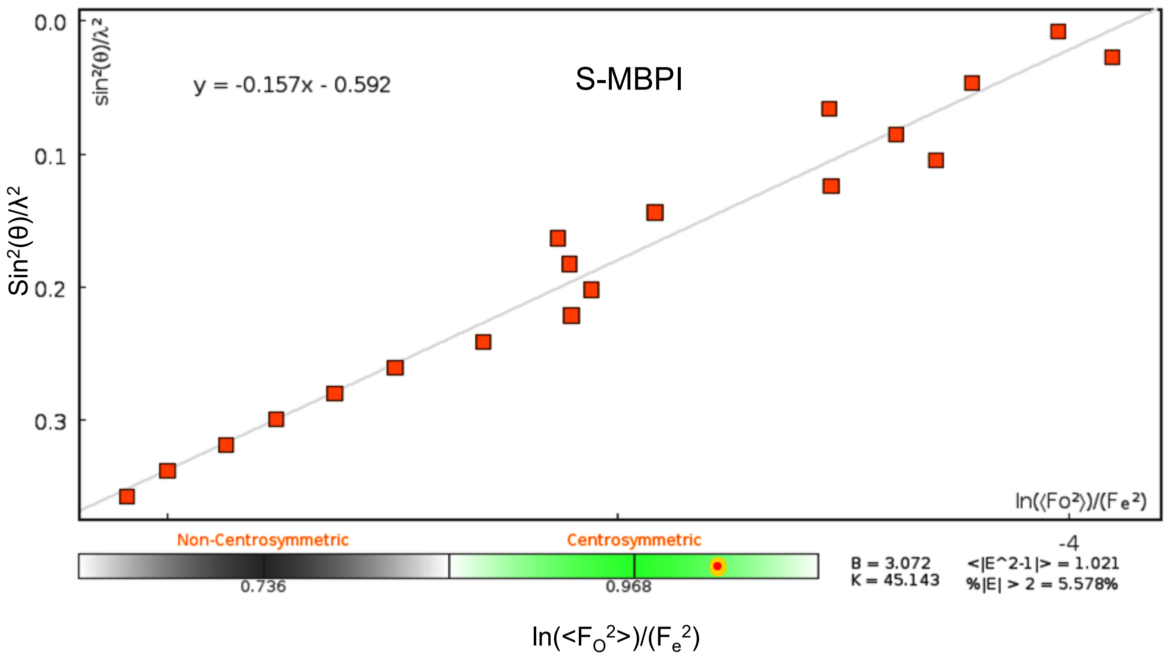

Supplementary Figure 3 | a, b, Wilson statistics obtained during the data reduction of 298 K single-crystal X-ray diffraction data for S-NPB (**a**) and S-MBPI (**b**). The statistics give $<E^2-1>$ values of 0.808 for S-NPB (i.e., corresponding to a noncentrosymmetric space group) and 1.021 for S-MBPI (i.e., corresponding to a centrosymmetric space group). Note that the inorganic framework comprising the heavy Pb and Br/I atoms scatters X-rays more strongly than the organic framework, and the Wilson statistics therefore preferentially reflect the inorganic framework symmetry. The Wilson statistics results for R-NPB vs. R-MBPI are analogous to the above.

a

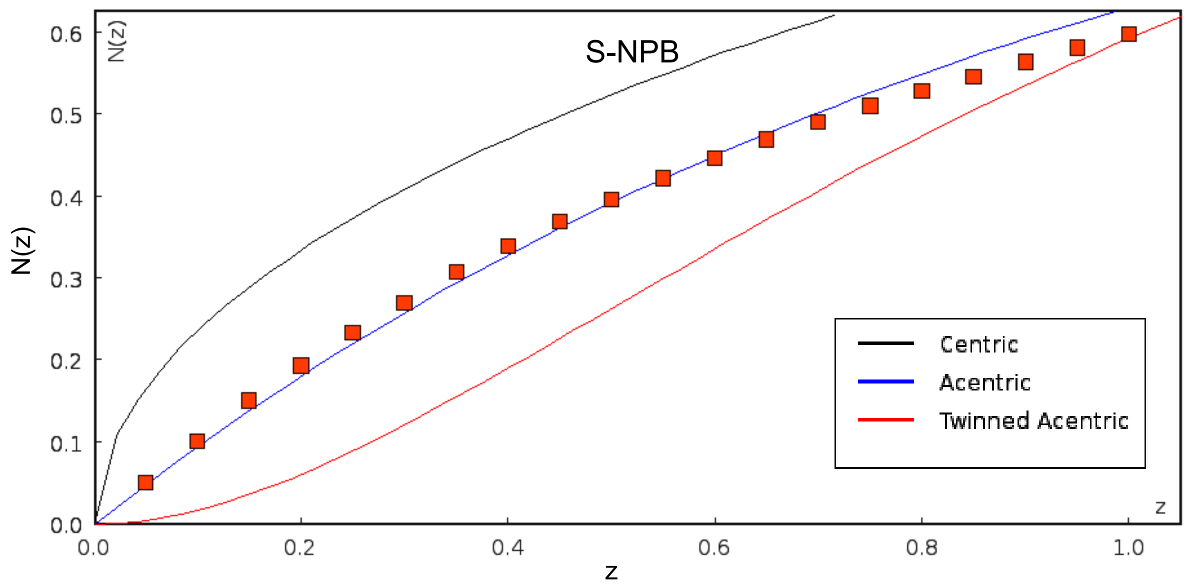

b

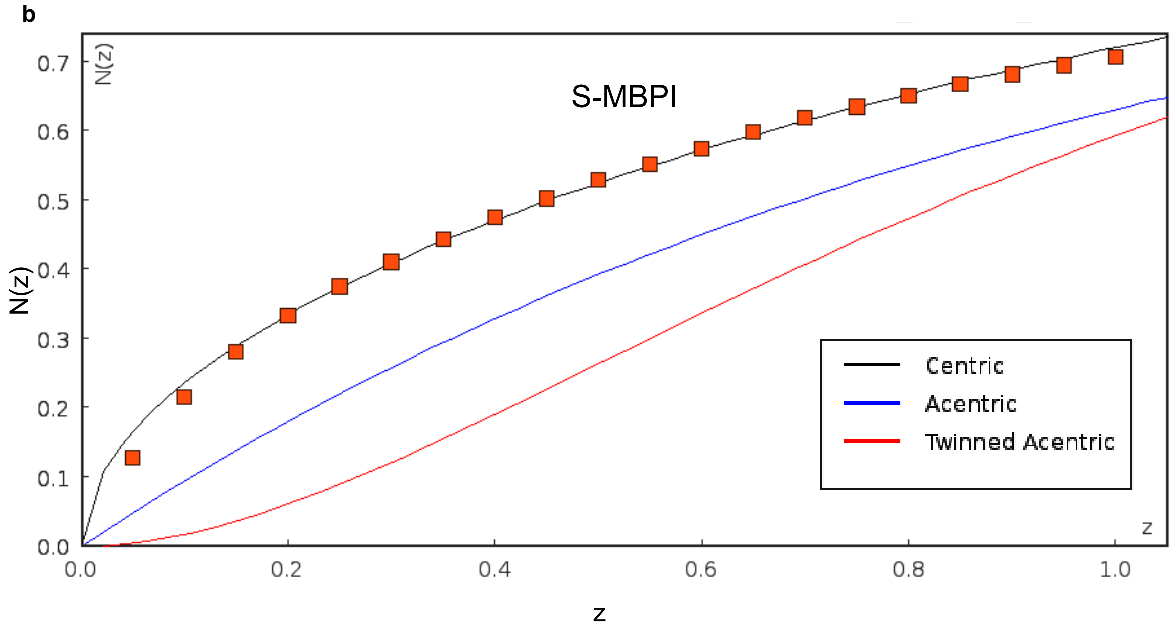

Supplementary Figure 4 | a,b, The cumulative intensity distribution of the 298 K single-crystal X-ray diffraction data for S-NPB (**a**) and S-MBPI (**b**). The X-ray reflections are fit to an acentric distribution in S-NPB and to a centric distribution in S-MBPI. Note that the inorganic framework comprising the heavy Pb and Br/I atoms scatters X-rays more strongly than the organic framework, and the cumulative intensity distribution therefore preferentially reflects the inorganic framework symmetry. The cumulative intensity distributions for R-NPB vs. R-MBPI are analogous to the above.

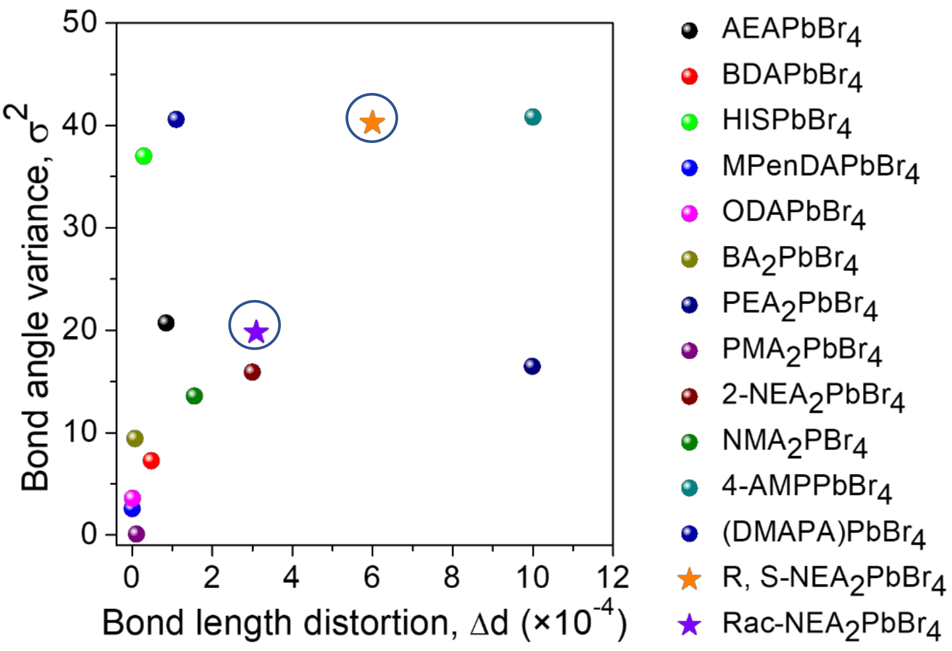

Supplementary Figure 5 | A plot of bond length distortion (Δd) vs. bond angle variance (σ^2), computed based on single-crystal X-ray structures of various 2D lead bromide based HOIPs reported in the literature including the racemic-, R- and S-NPB studied here. **AEA**: 3-(2-ammonioethyl)anilinium[1]; **BDA**: 1,4-butyldiammonium[1]; **HIS**: histammonium[1]; **MPenDA**: 2-methyl-1,5-pentanediammonium[1]; **ODA**: 1,8-diammoniooctane[1]; **BA**: n-butylammonium[1]; **PEA**: phenethylammonium[2]; **PMA**: phenylmethylammonium[3]; **2-NEA**: 2-(2-naphthyl)ethanammonium[3]; **NMA**: 1-(2-naphthyl)methanammonium[3]; **4-AMP**: 4-(aminomethyl)-piperidine[4]; **DMAPA**: 3-(dimethylamino)-1-propylamine[5]; **R/S/Rac-NEA**: R-/S-/rac- 1-(1-naphthyl)ethylammonium (this work).

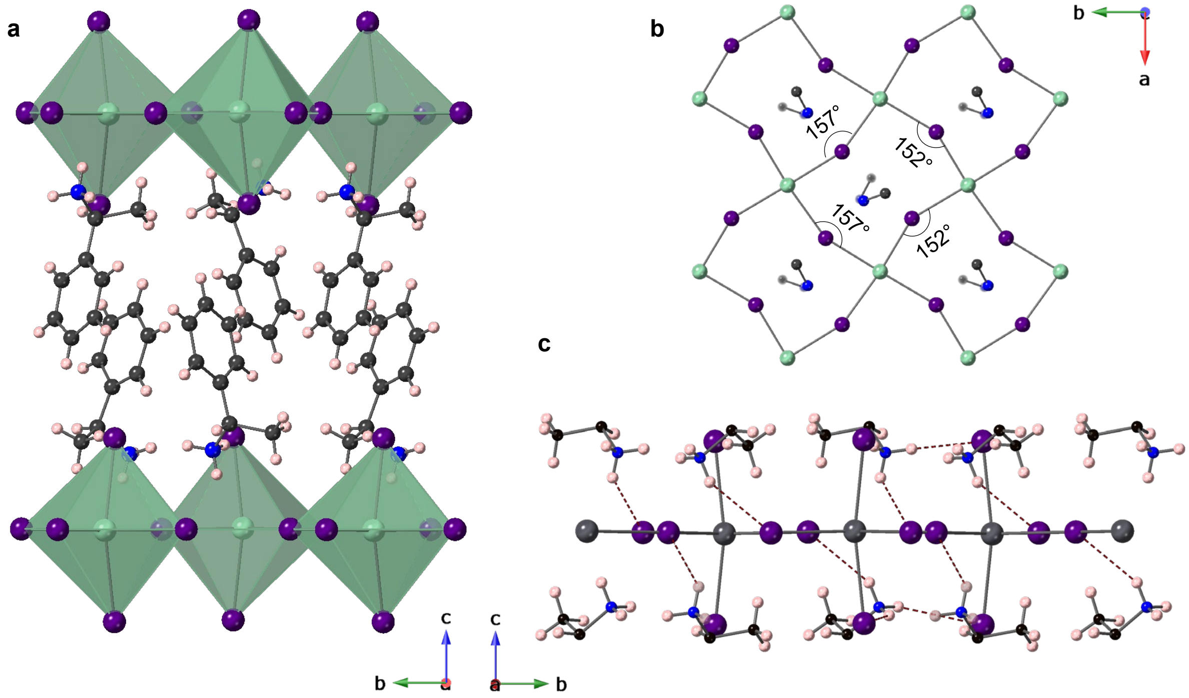

Supplementary Figure 6 | a, Schematic representation of single-crystal X-ray structure of S-MBPI at 298 K. **b**, In-plane view of a single perovskite $[PbI_4]^{2-}$ layer showing two equatorial Pb-I-Pb angles of 152° and 157°. Also shown are the organic terminal -CH-NH_3^+ groups represented as solid and shaded dumbbells for the upper and lower organic layers, respectively. **c**, Hydrogen bonding interactions of equatorial I atoms with -NH_3^+ groups, with alternating H…I contacts of 2.838 Å and 3.088 Å. Axial I atoms are omitted in (**b**) for clarity. Pb, I, C, N, H are denoted as green, purple, black, blue and pink spheres, respectively. Refer to **Supplementary Table 2** for complete list of hydrogen bonding parameters.

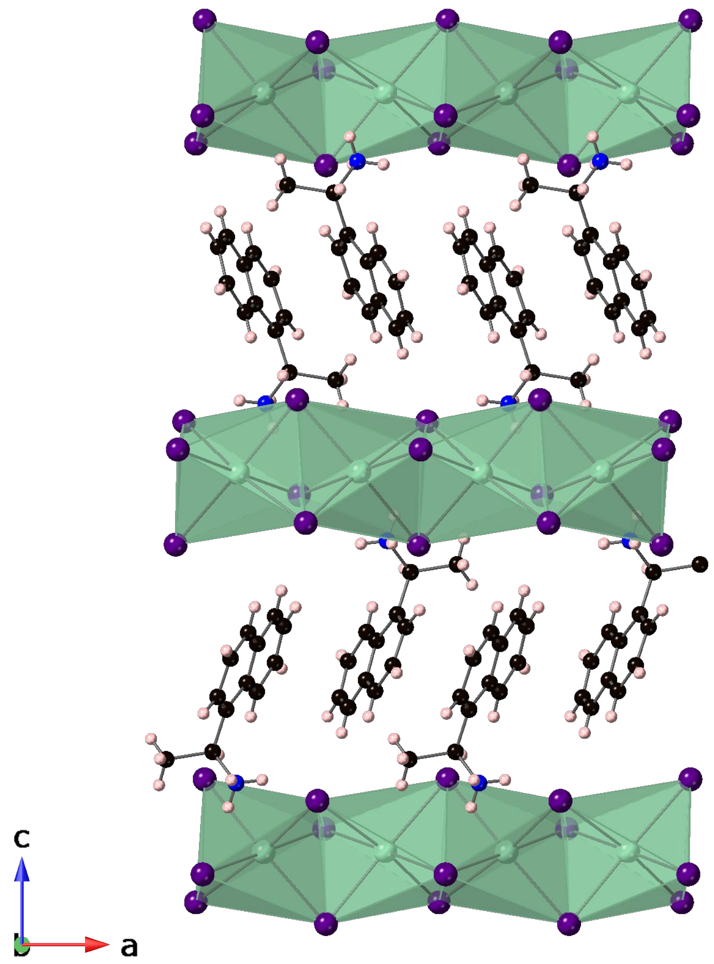

Supplementary Figure 7 | Schematic single-crystal X-ray structure of 1D S-$NEA_2Pb_2I_6$ at 298 K. 1D chains of face-sharing PbI_6 octahedra are separated by NEA^+ (1-(1-napthyl)ethylammonium) cations. Pb, I, C, N, H are denoted as green, purple, black, blue, and pink spheres, respectively.

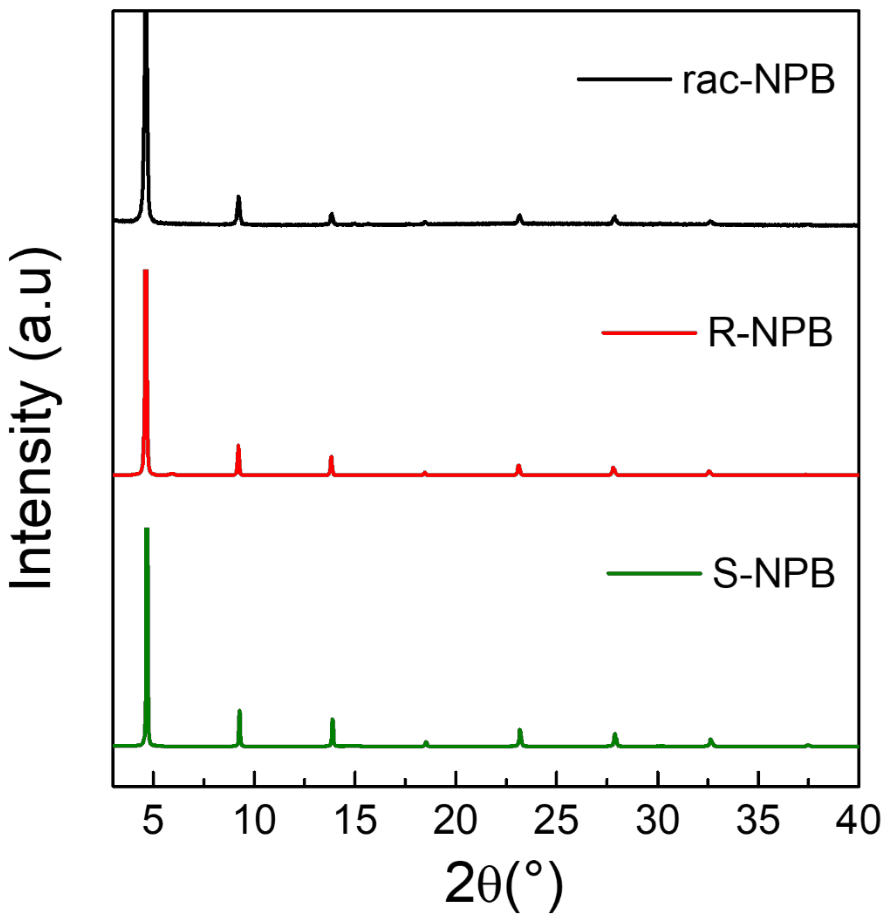

Supplementary Figure 8 | Powder X-ray diffraction patterns of spin-coated thin films of racemic-, R- and S-NPB.

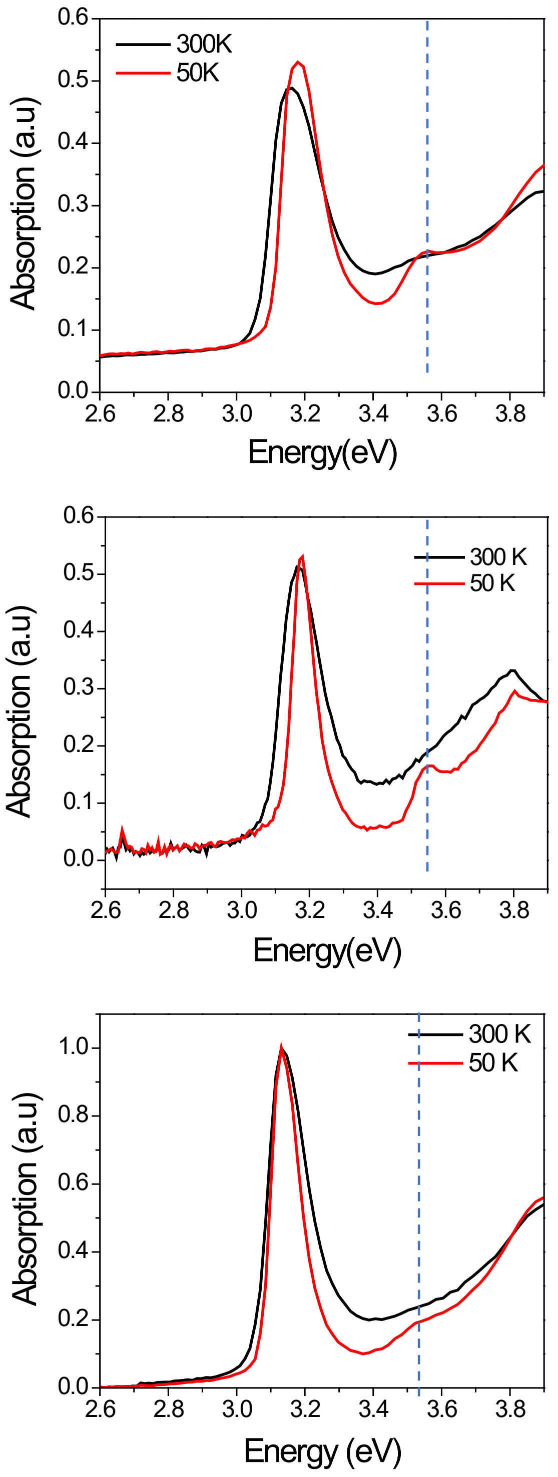

Supplementary Figure 9 | a-c, Thin-film linear absorption spectra of R-NPB (**a**), S-NPB (**b**) and racemic-NPB (**c**) at 300 K and 50 K. The dotted lines indicate the continuum band-edges, which are at 3.54(2) eV for the R- and S-NPB and 3.52(2) eV for the racemic-NPB. The band-edge energy is estimated from the photon energy of the maximum (indicated by the dashed lines) in the step-like feature preceding the excitonic band in the 50 K spectra.

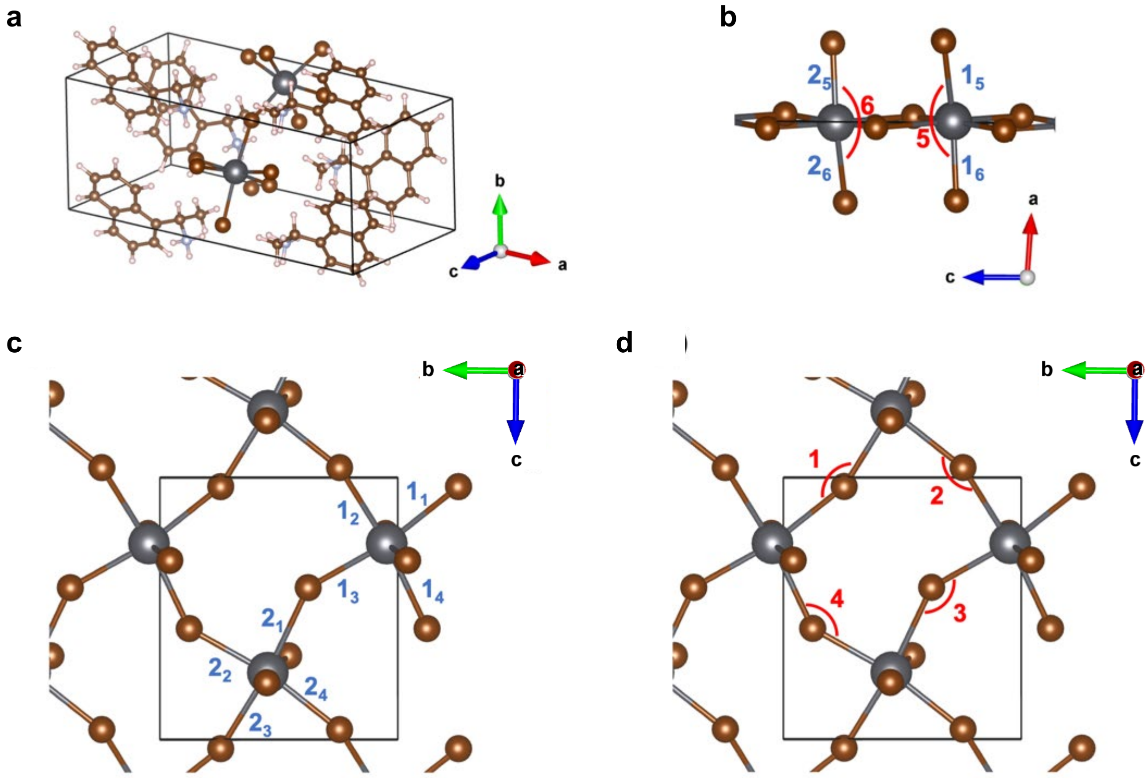

Supplementary Figure 10 | a-d, Illustration of the bond length and bond angle labels used in geometry comparison between experimental and relaxed structures. Relaxed S-NPB structure serves as the example here. **a**, DFT-PBE+TS relaxed S-NPB unit cell. **b**, Side view of the inorganic layer along *b*-axis. **c**,**d**, Top-views of the inorganic layer along *a*-axis. The labels of bond lengths are colored in blue and bond angles are colored in red.

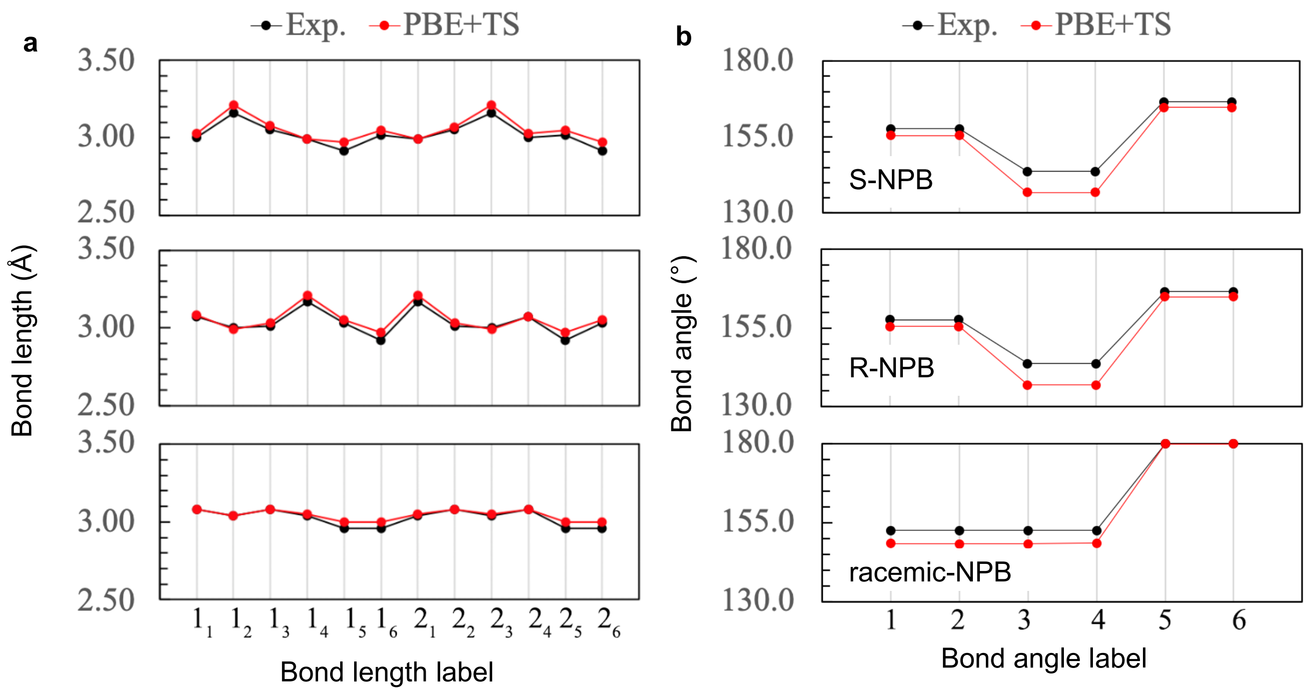

Supplementary Figure 11 | a, b, Comparison of (**a**) bond lengths and (**b**) bond angles between experimental (black data points) and DFT-PBE+TS relaxed (red data points) structures of R-, S-, and racemic-NPB. The label notations in (**a**) and (**b**) are the same as in **Supplementary Figure 10.** The agreement between theory and experiment is very good.

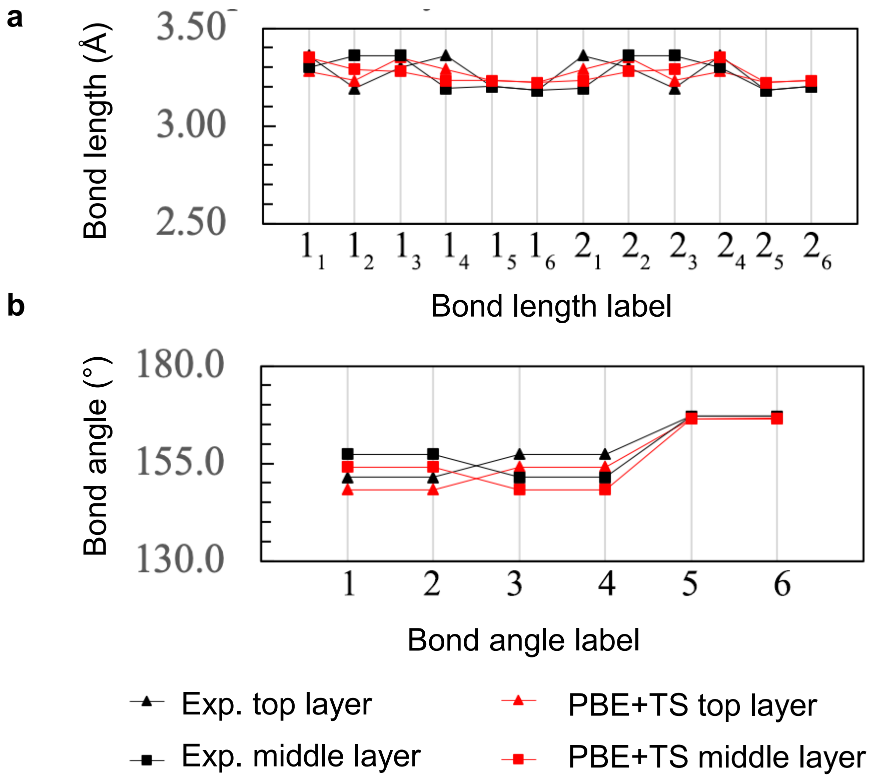

Supplementary Figure 12 | a, b, Geometry comparisons of (**a**) bond lengths and (**b**) bond angles in the perovskite layer between the experimental and DFT-PBE+TS relaxed structures of S-MBPI. The two inorganic layers in the unit cell of S-MBPI are labelled as 'top layer' and 'middle layer'. The label notations in (**a**) and (**b**) are the same as in **Supplementary Figure 10.** The agreement between theory and experiment is very good.

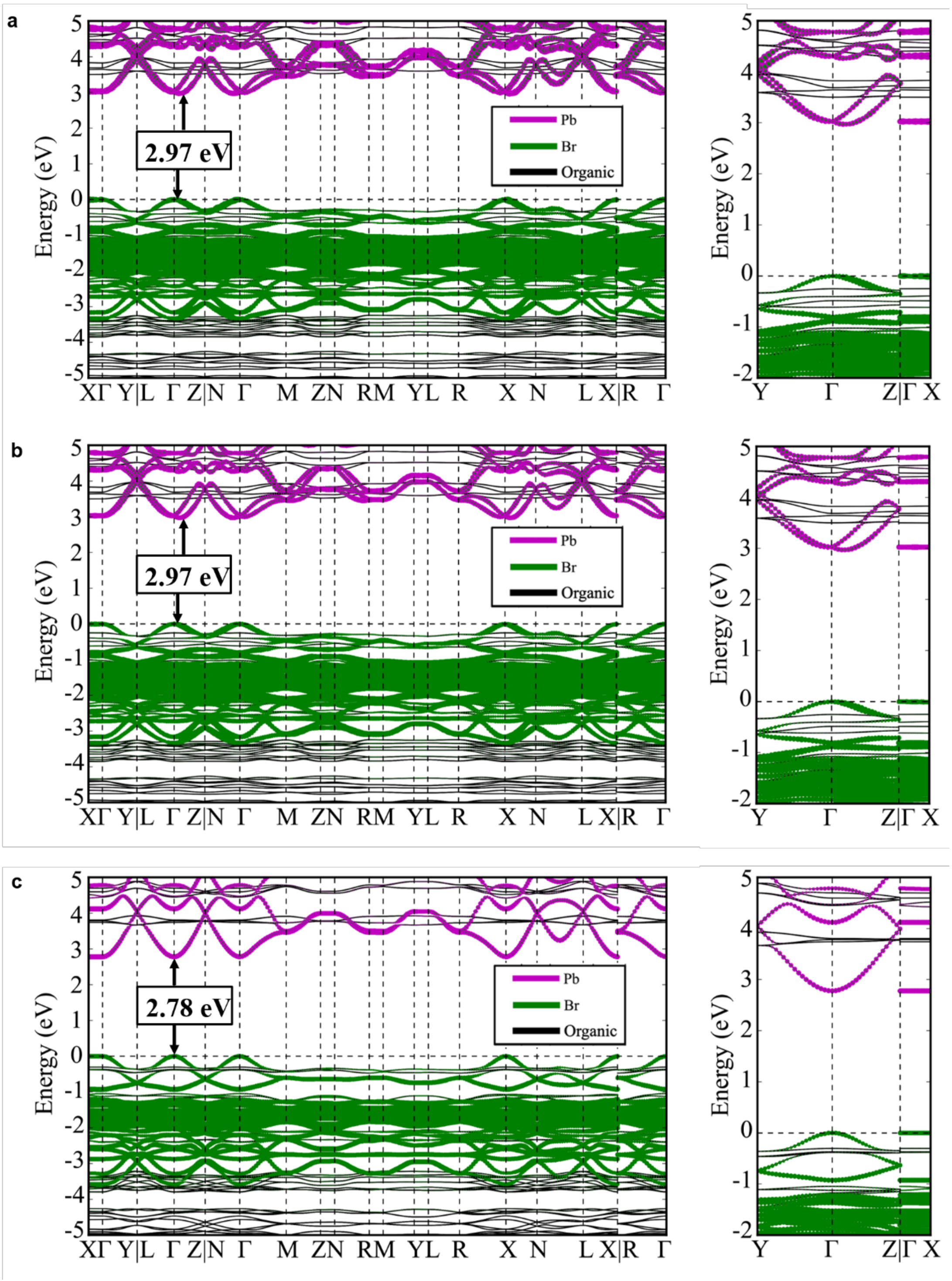

Supplementary Figure 13 | a-c, DFT+HSE06+SOC electronic band structures of (**a**) S-NPB, (**b**) R-NPB and (**c**) racemic-NPB for all the k-paths. The contributions of Pb (purple), Br (green) and organic-derived states (black) to the band structure are indicated. CB minima and VB maxima are pointed out by black arrows and the size of the band gap is shown for each structure.

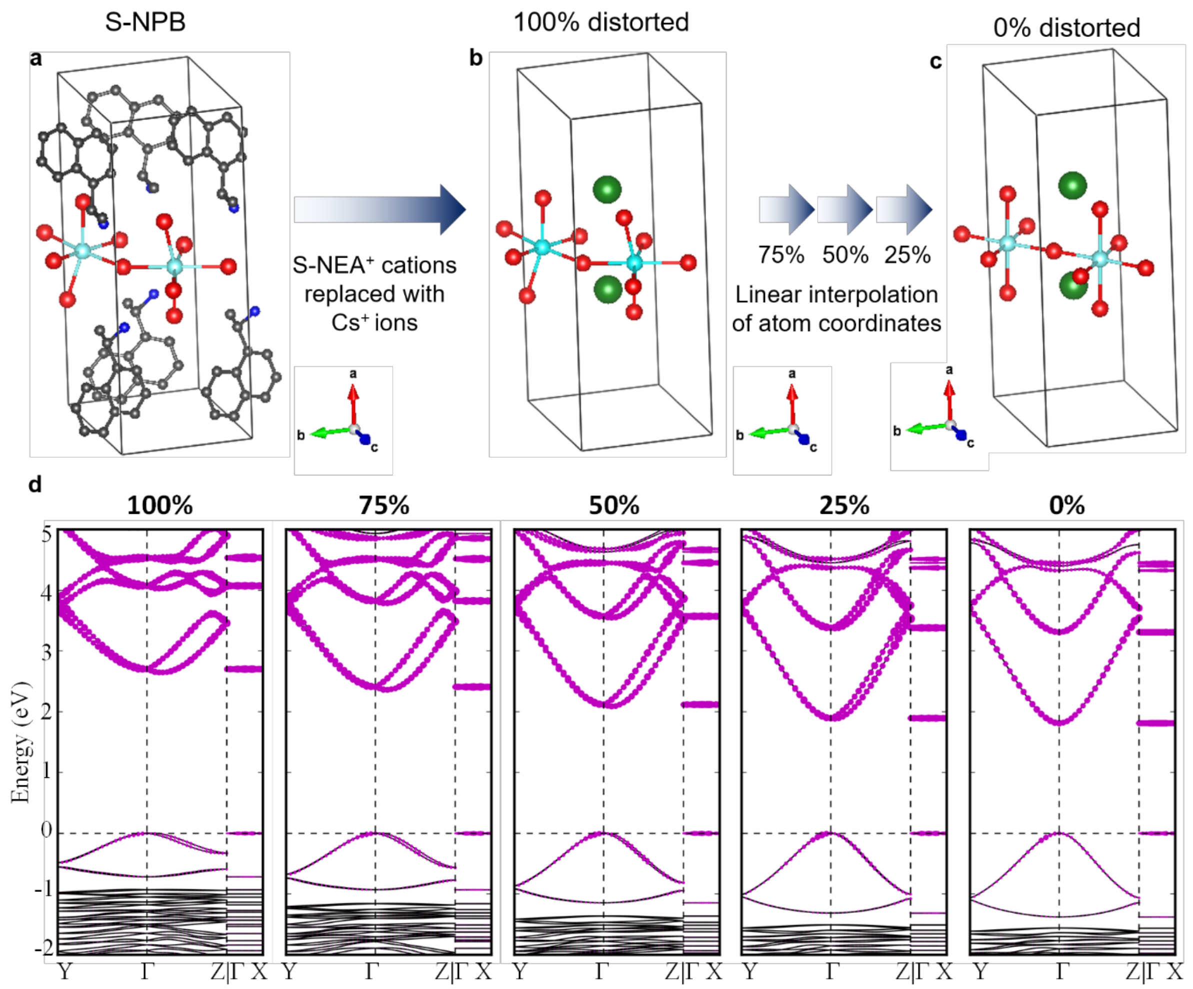

Supplementary Figure 14 | a, Original S-NPB relaxed structure. **b,** S-NEA$^+$ cations in (**a**) are replaced with Cs$^+$ ions maintaining the original octahedral distortions (denoted as 100%). **c**, An ideal structure where the octahedral distortions in (**b**) are artificially removed (denoted as 0%). By interpolation of the atomic coordinates between the ideal and the distorted structure, intermediate structures with gradually decreasing distortions (75%, 50%, 25%) are generated similarly. **d**, HSE06+SOC band structures calculated for the as-generated structures corresponding to 100%, 75%, 50%, 25%, and 0% octahedral distortions with respect to the original S-NPB structure. Note that the RD splitting along Γ-Z path gradually disappears upon going from 100% to 0% octahedral distortions. Cyan, red, black, blue, and green spheres denote Pb, Br, C, N, and Cs atoms, respectively.

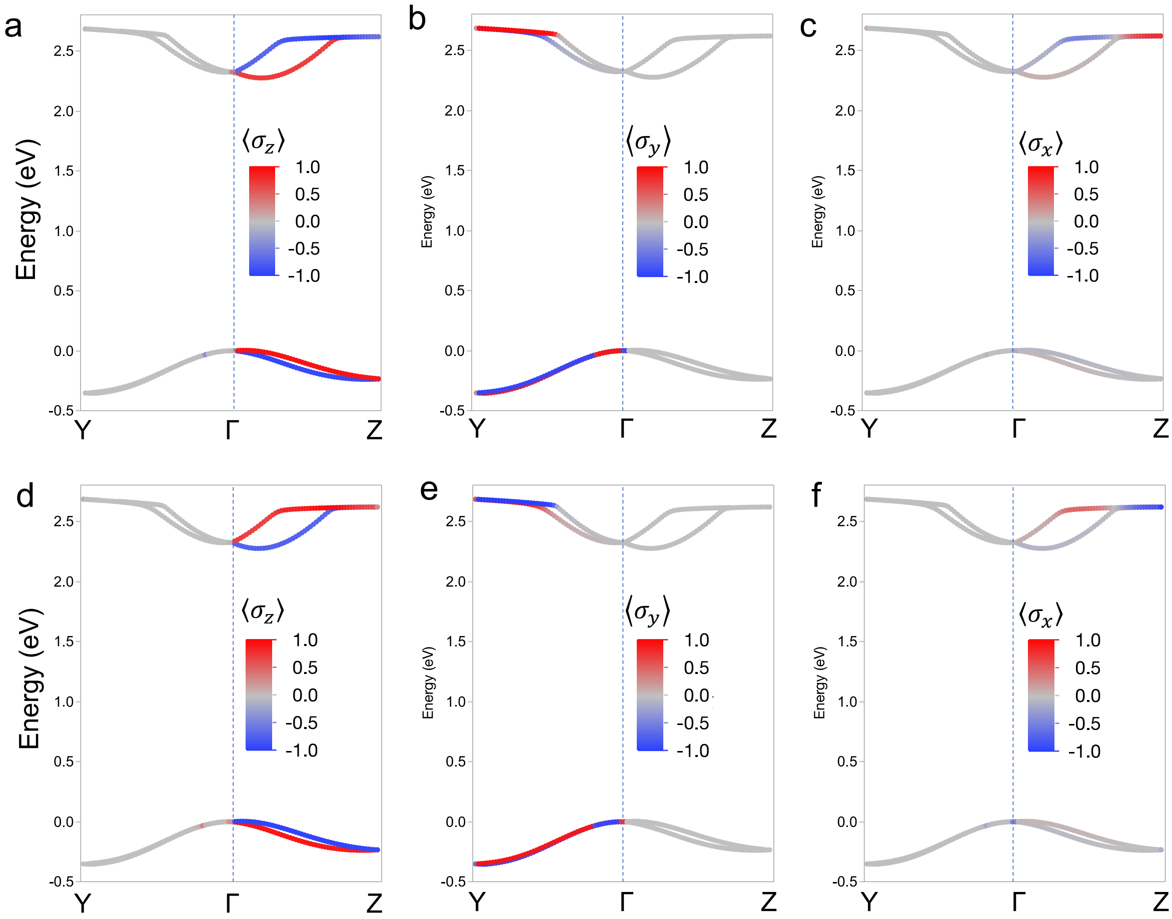

Supplementary Figure 15 | PBE+SOC band structures of (**a-c**) R-NPB and (**d-f**) S-NPB showing the spin polarization of frontier conduction and valence bands. The color bars give the magnitudes of $\langle\sigma_x\rangle$, $\langle\sigma_y\rangle$, and $\langle\sigma_z\rangle$, which are the expectation values of the Pauli spin matrices, σ_i (i=x,y,z). Here, x and y point along the two in-plane directions of the perovskite layer and z points along the layer-stacking direction. Note that along the spin splitting direction (i.e., the Γ-Z path), the dominant spin component is σ_z (**a, d**); the other two components are vanishingly small in line with a 1D RD picture, as previously noted for a related 2D HOIP, PMA_2PbCl_4.[6] Notably, owing to opposite structural chiralities of perovskite layers in R- and S-NPB, the spin polarization of conduction and valence spin subbands is opposite between R-NPB (**a**) and S-NPB (**d**), as clearly seen from the opposite signs of the respective σ_z components.

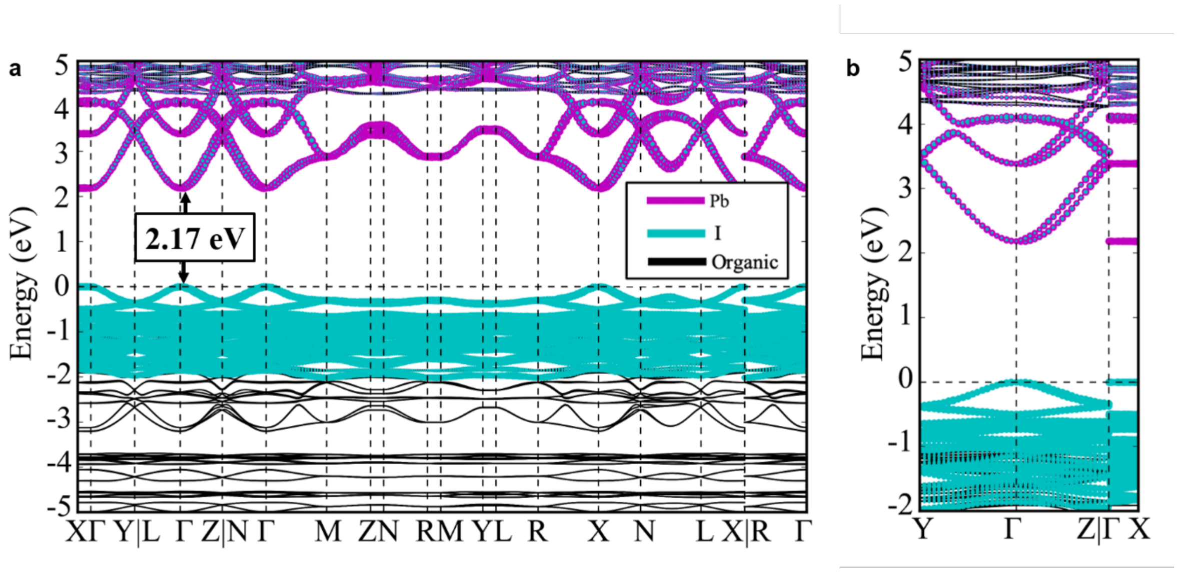

Supplementary Figure 16 | The DFT+HSE06+SOC (**a**) full band structure of S-MBPI and (**b**) selected extract along k-paths Y-Γ, Γ-Z and Γ-X showing a much lower RD splitting in this compound relative to R- and S-NPB. The contributions of Pb (purple), I (cyan) and of the organic component (black) to the band structure are indicated. CB minima and VB maxima are pointed out by black arrows and the size of the band gap is given.

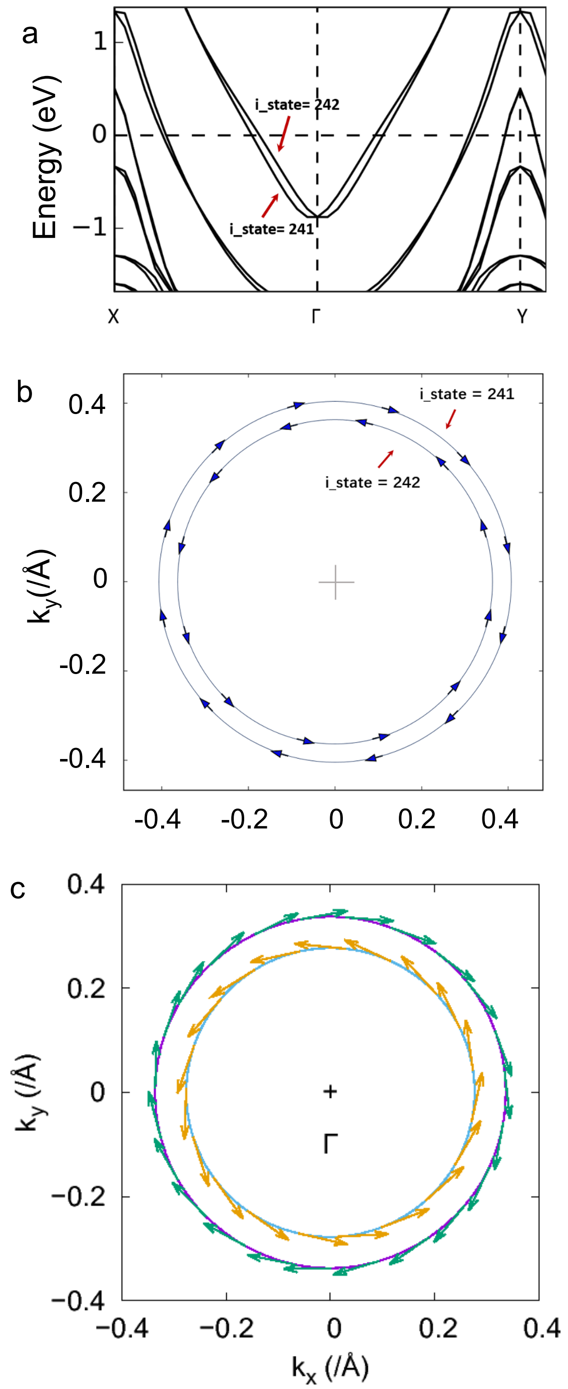

Supplementary Figure 17 | a, Rashba splitting in the LDA band structure of the Au(111) surface. **b,** spin texture of the 241st and 242nd band at the Fermi surface, obtained from FHI-aims. **c,** spin texture of the 55th and 56th bands (corresponding to the 241st and 242nd bands in (**b**)) at the Fermi surface, reported using the OpenMX code (references 7-9).

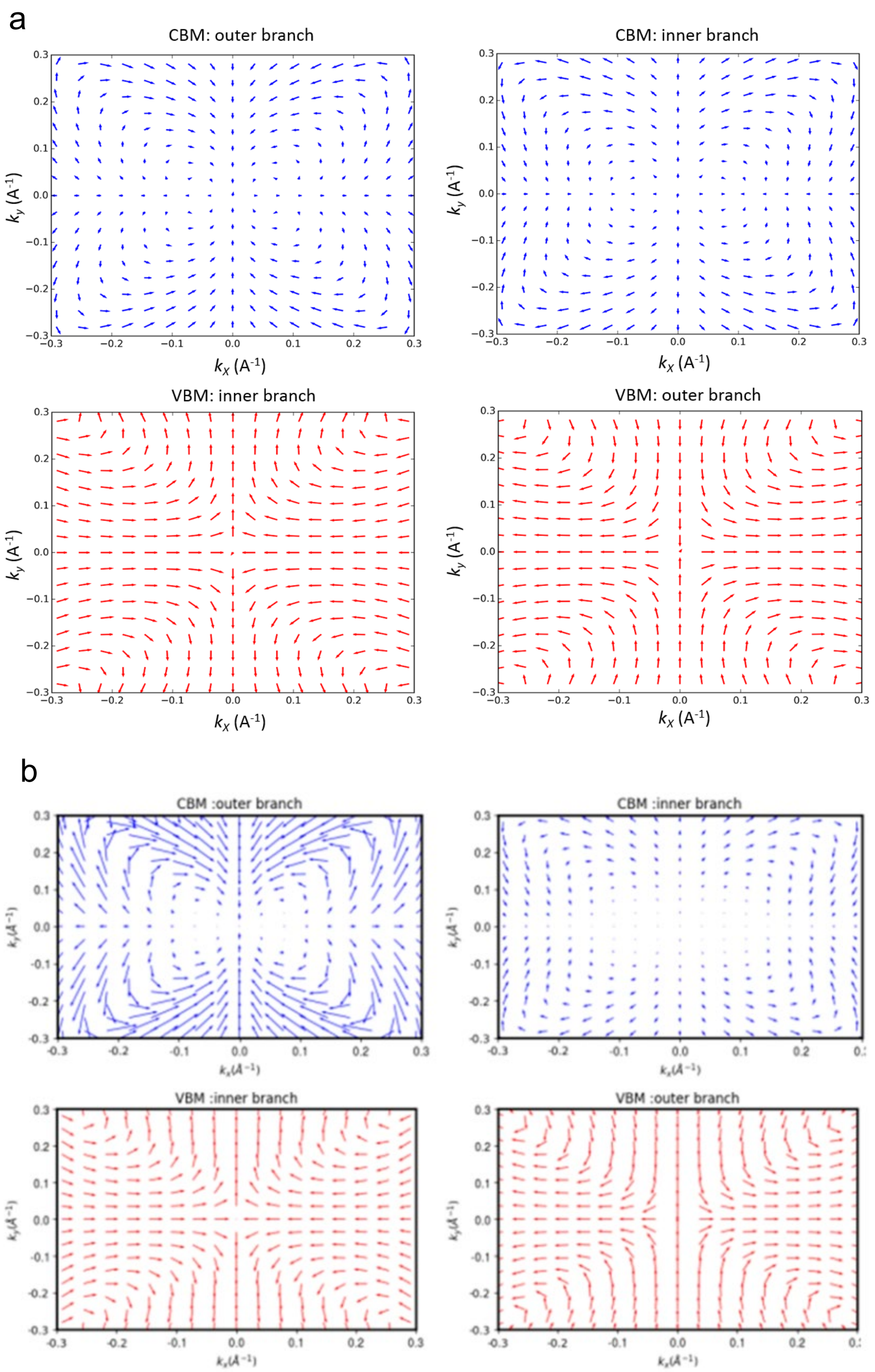

Supplementary Figure 18 | a, The spin textures of the inner and outer branches of the valence (red) and conduction (blue) bands of (4-BrBzA)$_2$PbI$_4$. Arrow length denotes the magnitude of the spin polarizations in the $k_x - k_y$ plane. **b,** The corresponding spin textures reported by reference 10. Both results were calculated using the PBE functional. Panel (**b**) is adapted from reference 10. © 2020, American Chemical Society.

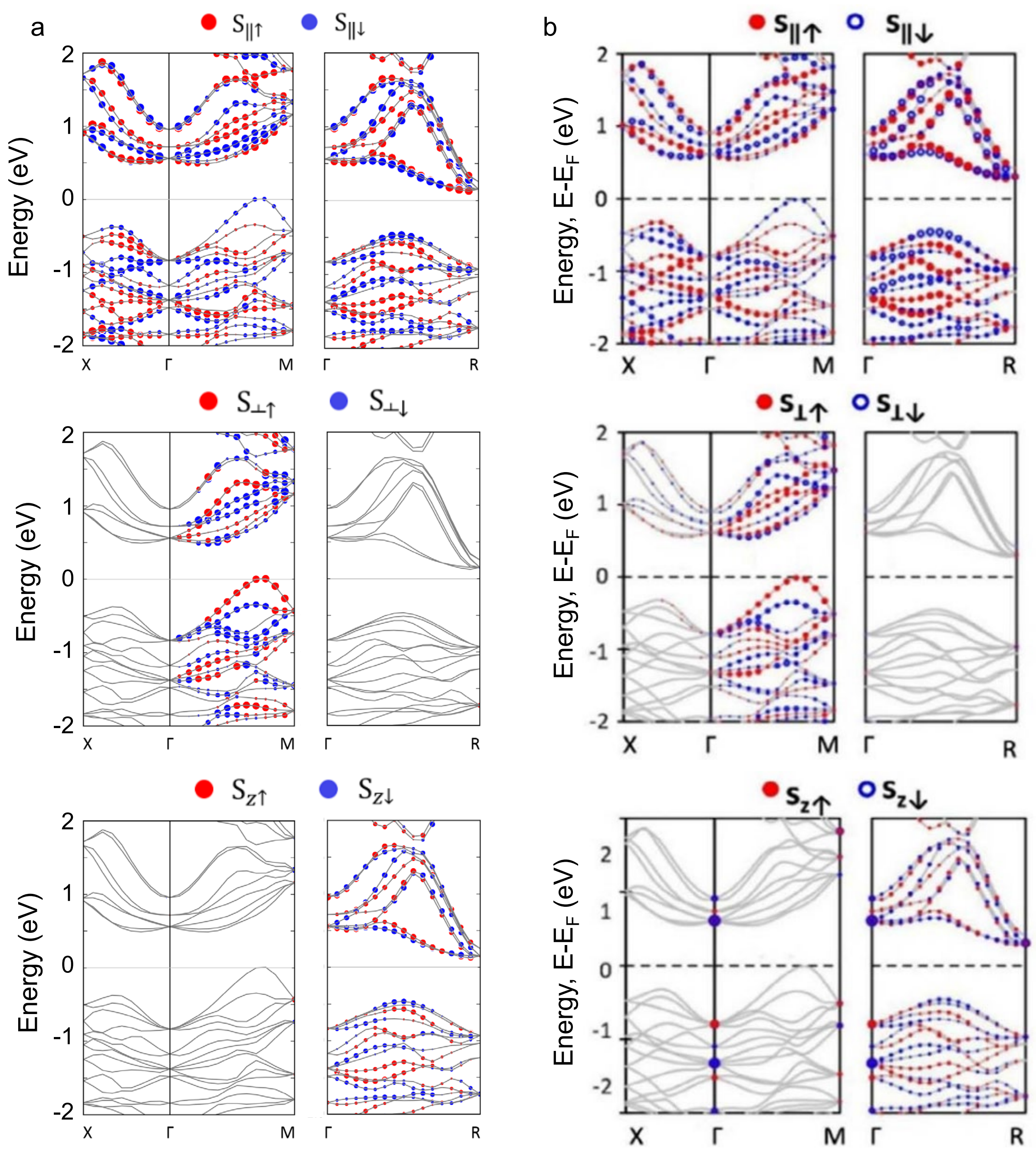

Supplementary Figure 19 | a, The LDA spin textures of bulk IrBiSe, calculated using FHI-aims. $S_{\|}$ is the spin projection along the *k*-direction; $S_{\perp}$ is the spin projections perpendicular to the *k*-direction; S_z is the *z* component of the spin polarization, which is also along k_z-direction. The symbol sizes are proportional to the magnitude of the spin polarizations. **b,** The corresponding spin texture results in reference 11. Note that the net spin polarizations for all the degenerate bands at Γ point should be exactly zero. The visually red and blue dots at Γ in the right panel are simply leftovers due to the plotting style (each red/blue dot is overwritten by the blue/red dot of the other degenerate band, and their sum is zero). Panel (**b**) is adapted from reference 11. © 2020, WILEY-VCH.

Supplementary Tables

Supplementary Table 1 | Crystallographic and structural refinement data for R-, S- and racemic-NPB at 298 K

Empirical formula	R-$C_{24}H_{28}N_2PbBr_4$	S-$C_{24}H_{28}N_2PbBr_4$	Racemic-$C_{24}H_{28}N_2PbBr_4$
Formula weight	871.31	871.31	871.31
Temperature/K	298	298	298
Crystal system	monoclinic	monoclinic	monoclinic
Space group	$P2_1$	$P2_1$	$P2_1/c$
a/Å	8.7569(2)	8.7537(2)	19.2528(9)
b/Å	7.9611(2)	7.9550(2)	8.0769(4)
c/Å	19.5188(6)	19.5038(5)	8.7280(5)
α/°	90	90	90
β/°	93.773(2)	93.806(2)	90.281(3)
γ/°	90	90	90
Volume/Å^3	1357.78(6)	1355.17(5)	1357.21(12)
Z	2	2	2
ρ_{calc}g/cm^3	2.131	2.135	2.132
μ/mm^{-1}	12.113	12.136	12.118
F(000)	816.0	816.0	816.0
Radiation	MoKα (λ = 0.71073)	MoKα (λ = 0.71073)	MoKα (λ = 0.71073)
2Θ range for data collection/°	4.182 to 61.868	4.186 to 52.728	5.47 to 53.128
Reflections collected	17623	18140	2783
Independent reflections	6685 [R_{int} = 0.0297, R_{sigma} = 0.0336]	5520 [R_{int} =0.0477, R_{sigma} = 0.0368]	2783 [R_{int} = 0.0436, R_{sigma} = 0.0395]
Data/restraints/parameters	6685/1/284	5520/1/236	2783/0/133
Goodness-of-fit on F^2	1.000	1.045	1.100
Final R indexes [I>=2σ (I)]	R_1 = 0.0307, wR_2 = 0.0797	R_1 = 0.0472, wR_2 = 0.1167	R_1 = 0.0483, wR_2 = 0.1227
Final R indexes [all data]	R_1 = 0.0362, wR_2 = 0.0812	R_1 = 0.0563, wR_2 = 0.1248	R_1 = 0.0581, wR_2 = 0.1271
Largest diff. peak/hole / e Å^{-3}	1.62/-1.36	2.03/-2.13	3.64/-1.83
Flack parameter	-0.018(7)	-0.023(11)	

Supplementary Table 2 | Experimental hydrogen bonding parameters based on 298 K single crystal X-ray structures for various 2D HOIPs studied in this work. The subscripts 'eq' and 'ax' denote equatorial and axial, respectively.

Compound	H-bond	length (Å)	angle at H (°)
	N-H---Br_{eq}	2.922	155.774
	N-H---Br_{eq}	2.865	139.996
	N-H---Br_{ax}	2.483	173.457
**R-NPB**	N-H---Br_{ax}	2.587	172.108
	N-H---Br_{ax}	2.686	170.925
	N-H---Br_{ax}	2.493	157.262
	C-H---Br_{eq}	2.829	160.034
	N-H---Br_{eq}	2.809	146.426
	N-H---Br_{eq}	2.935	150.543
	N-H---Br_{ax}	2.659	176.230
**S-NPB**	N-H---Br_{ax}	2.587	176.575
	N-H---Br_{ax}	2.506	151.769
	N-H---Br_{ax}	2.465	174.464
	C-H---Br_{eq}	2.822	159.294
	N-H---Br_{eq}	2.597	155.541
**Racemic-NPB**	N-H---Br_{ax}	2.468	152.393
	N-H---Br_{ax}	2.659	157.790
	N-H---Br_{eq}	3.088	132.054
	N-H---Br_{eq}	2.838	151.015
**S-MBPI**	N-H---Br_{ax}	2.850	159.008
	N-H---Br_{ax}	2.641	155.389
	N-H---Br_{ax}	2.776	137.539
	N-H---Br_{ax}	3.010	148.490

Supplementary Table 3 | Global space group, inorganic layer symmetry, and computed distortion parameters based on single-crystal structures of racemic-/R-/S-NPB and R-/S-MBPI.

Compound	*Space group*	*Inorganic symmetry (PLATON)*	*σ^2 (deg^2)*	*Δd (10^{-4})*	*Axial Br-Pb-Br bond angle*	*Equatorial Pb-Br-Pb bond angle(s)*
Racemic-NPB	$P2_1/c$	$P2_1/c$ (centro)	19.8	3.1	180°	153°
R/S-NPB	$P2_1$	$P2_1$ (chiral)	40.26	6	166°	143°, 157°
R/S-MBPI	$P2_12_12_1$	*Pnma* (centro)	20.08	5.6	167°	151°, 157°

Supplementary Table 4 | Crystallographic and structural refinement data for S-MBPI at 100, 200 and 298 K

Empirical formula	S-$C_{16}H_{24}N_2PbI_4$	S-$C_{16}H_{24}N_2PbI_4$	S-$C_{16}H_{24}N_2PbI_4$
Formula weight	959.16	959.16	959.16
Temperature/K	100	200	298
Crystal system	orthorhombic	orthorhombic	orthorhombic
Space group	$P2_12_12_1$	$P2_12_12_1$	$P2_12_12_1$
a/Å	28.6004(6)	8.8697(2)	8.9034(2)
b/Å	9.2078(2)	28.7060(8)	28.8647(7)
c/Å	8.8408(2)	9.2496(3)	9.3127(2)
α/°	90	90	90
β/°	90	90	90
γ/°	90	90	90
Volume/Å^3	2328.19(9)	2355.07(11)	2393.31(9)
Z	4	4	4
ρ_{calc}g/cm^3	2.736	2.705	2.662
μ/mm^{-1}	12.552	12.409	12.210
F(000)	1712.0	1712.0	1712.0
Radiation	MoKα (λ = 0.71073)	MoKα (λ = 0.71073)	MoKα (λ = 0.71073)
2Θ range for data collection/°	4.648 to 52.74	4.626 to 52.736	4.596 to 52.734
Reflections collected	17922	12303	14448
Independent reflections	4725 [R_{int} = 0.0251, R_{sigma} = 0.0194]	4701 [R_{int} = 0.0199, R_{sigma} = 0.0226]	5731 [R_{int} = 0.0237, R_{sigma} = 0.0255]
Data/restraints/parameters	4725/0/212	4701/0/212	5731/0/212
Goodness-of-fit on F^2	1.123	1.004	1.040
Final R indexes [I>=2σ (I)]	R_1 = 0.0191, wR_2 = 0.0460	R_1 = 0.0194, wR_2 = 0.0446	R_1 = 0.0313, wR_2 = 0.0722
Final R indexes [all data]	R_1 = 0.0203, wR_2 = 0.0465	R_1 = 0.0228, wR_2 = 0.0455	R_1 = 0.0391, wR_2 = 0.0747
Largest diff. peak/hole / e Å^{-3}	1.41/-0.98	1.54/-0.58	0.93/-1.48
Flack parameter	0.024(4)	0.009(6)	0.024(8)

Supplementary Table 5 | Crystallographic and structural refinement data for S-$NEA_2Pb_2I_6$ (S-NPI) at 298 K

Empirical formula	S-$C_{24}H_{28}N_2Pb_2I_6$
Formula weight	1520.26
Temperature/K	298
Crystal system	orthorhombic
Space group	$P2_12_12_1$
a/Å	8.1000(3)
b/Å	8.4632(3)
c/Å	25.3124(9)
α/°	90
β/°	90
γ/°	90
Volume/Å^3	1735.21(11)
Z	2
ρ_{calc}g/cm^3	2.910
μ/mm^{-1}	15.043
F(000)	1336.0
Crystal size/mm^3	0.159 × 0.075 × 0.046
Radiation	MoKα (λ = 0.71073)
2Θ range for data collection/°	5.28 to 52.766
Index ranges	$-10 \leq h \leq 10$, $-10 \leq k \leq 10$, $-31 \leq l \leq 30$
Reflections collected	12321
Independent reflections	3503 [R_{int} = 0.0240, R_{sigma} = 0.0367]
Data/restraints/parameters	3503/0/157
Goodness-of-fit on F^2	1.145
Final R indexes [I>=2σ (I)]	R_1 = 0.0228, wR_2 = 0.0504
Final R indexes [all data]	R_1 = 0.0264, wR_2 = 0.0586
Largest diff. peak/hole / e Å^{-3}	0.59/-0.92
Flack parameter	0.014(9)

Supplementary Table 6 | Comparison of lattice parameters and unit cell volumes between experimental and relaxed structures of S-, R-, and racemic-NPB, as well as S-MBPI. The experimental axes were reordered to fit the convention chosen for the theoretical axes.

Compound		*a* (Å)	*b* (Å)	*c* (Å)	*α* (°)	*β* (°)	*γ* (°)	*V* (Å^3)
	Exp.	19.5	7.96	8.75	90.0	93.8	90.0	1355
**S-NPB**	PBE+TS	19.27	7.79	8.76	90.0	95.3	90.0	1309
	Deviation	-1.19%	-2.18%	0.11%		1.5		-3.5%
	Exp.	19.52	7.96	8.76	90.0	93.77	90.0	1358
**R-NPB**	PBE+TS	19.27	7.79	8.76	90.0	95.4	90.0	1309
	Deviation	-1.29%	-2.18%	0%		1.63		-3.7%
	Exp.	19.25	8.08	8.73	90.0	90.3	90.0	1357
**Racemic-NPB**	PBE+TS	19.28	7.90	8.75	90.0	92.0	90.0	1332
	Deviation	0.15%	-2.22%	0.24%		1.7		-1.89%
	Exp.	28.87	8.90	9.31	90.0	90.0	90.0	2393
**S-MBPI**	PBE+TS	28.83	8.81	9.19	90.0	90.0	90.0	2333
	Deviation	-0.13%	-1.07%	-1.36%				-2.54%

Supplementary Table 7 | Input geometry.in file of Au(111) surface corresponding to Supplementary Figure 17a,b.

lattice_vector	2.8830859661	0.0000000000	0.0000000000	
lattice_vector	-1.4415431023	2.4968258944	0.0000000000	
lattice_vector	0.0000000000	0.0000000000	50.0000000000	
atom_frac	0.333333353	0.666666693	0.423725433	Au
atom_frac	0.666666687	0.333333347	0.470806046	Au
atom_frac	0.000000000	0.000000000	0.517886658	Au
atom_frac	0.333333353	0.666666693	0.564967270	H

Supplementary Table 8 | Input geometry.in file of $(4\text{-BrBzA})_2PbI_4$ corresponding to Supplementary Figure 18a.

lattice_vector	8.6328001022	0.0000000000	0.0000000000	
lattice_vector	0.0000000000	8.9483003616	0.0000000000	
lattice_vector	-1.5433467726	0.0000000000	15.8576739839	
atom	0.578614831	8.447016716	15.850378990	Pb
atom	6.510838032	3.972866535	0.007294530	Pb
atom	1.930478096	8.089262962	3.201981544	I
atom	5.158975124	3.615113258	12.655692101	I
atom	0.656904459	8.734704971	12.715792656	I
atom	6.432549000	4.260554314	3.141880751	I
atom	3.833200932	2.201371431	0.105770685	I
atom	3.256252050	6.675521374	15.751902580	I
atom	8.122714996	1.229228020	0.229460537	I
atom	-1.033261418	5.703378201	15.628212929	I
atom	7.528899670	0.197757438	9.337791443	Br
atom	-0.439445734	4.671907425	6.519882202	Br
atom	5.186055183	3.527420044	8.733613968	Br
atom	1.903397918	8.001569748	7.124059677	Br
atom	6.997970581	7.758176327	2.694218636	N
atom	0.091482878	3.284026146	13.163455009	N
atom	2.780409336	4.662064552	2.476968765	N
atom	4.309043884	0.187914550	13.380704880	N
atom	6.366302490	8.831972122	4.838176250	C
atom	0.723150730	4.357821941	11.019497871	C
atom	5.758203030	7.937142849	5.764264584	C
atom	1.331250310	3.462992191	10.093409538	C
atom	6.082551003	8.026625633	7.132781982	C
atom	1.006902456	3.552475452	8.724891663	C
atom	7.001899719	0.044741500	7.532394886	C

atom	0.087553978	4.518891811	8.325278282	C
atom	7.532611847	0.948519826	6.603135109	C
atom	-0.443159044	5.422669888	9.254539490	C
atom	7.231693745	0.832191944	5.259990215	C
atom	-0.142240405	5.306342125	10.597683907	C
atom	6.027477264	8.742489815	3.352312326	C
atom	1.061975956	4.268339157	12.505360603	C
atom	2.847216606	3.677751541	4.717658043	C
atom	4.242236137	8.151902199	11.140015602	C
atom	3.408299208	2.451834202	5.161673069	C
atom	3.681154251	6.925984383	10.696001053	C
atom	4.132862091	2.380247831	6.320868969	C
atom	2.956591129	6.854398251	9.536805153	C
atom	4.292793274	3.597216845	7.072522640	C
atom	2.796660185	8.071367264	8.785151482	C
atom	3.721534252	4.724702358	6.644365311	C
atom	3.367919207	0.250552386	9.213308334	C
atom	2.998206377	4.832082272	5.472483158	C
atom	4.091246605	0.357931674	10.385190964	C
atom	2.075497150	3.758285999	3.422086000	C
atom	5.013956070	8.232436180	12.435587883	C
atom	7.850749969	8.056405067	2.804857969	H
atom	-0.761296391	3.582255363	13.052815437	H
atom	6.810063362	7.700755119	1.805491328	H
atom	0.279389977	3.226604939	14.052182198	H
atom	6.907192707	6.941661835	3.079306602	H
atom	0.182260633	2.467511892	12.778367043	H
atom	5.134750843	7.290198326	5.461350918	H
atom	1.954702497	2.816048145	10.396322250	H
atom	5.681096554	7.442471027	7.765248775	H

atom	1.408356905	2.968321085	8.092424393	H
atom	-0.522267103	1.642997503	6.897818089	H
atom	7.611721039	6.117147923	8.959855080	H
atom	7.643675804	1.410833716	4.629869938	H
atom	-0.554222107	5.884984016	11.227804184	H
atom	6.114563942	0.685171306	2.930640697	H
atom	0.974889278	5.159321308	12.927033424	H
atom	5.098641396	8.428072929	3.233791828	H
atom	1.990811706	3.953922749	12.623882294	H
atom	2.809207439	5.499258518	2.821666956	H
atom	4.280246258	1.025107741	13.036005974	H
atom	2.340891123	4.670001507	1.680929184	H
atom	4.748561859	0.195852026	14.176744461	H
atom	3.631511927	4.356023788	2.347252846	H
atom	3.457942009	8.830173492	13.510420799	H
atom	3.286421299	1.664240718	4.641620159	H
atom	3.803031921	6.138391018	11.216053009	H
atom	4.504052162	1.560583591	6.620975018	H
atom	2.585401297	6.034733295	9.236698151	H
atom	3.804540157	5.498775482	7.183209419	H
atom	3.284913063	1.024625659	8.674464226	H
atom	2.632024765	5.658269882	5.194799423	H
atom	4.457428932	1.184119582	10.662873268	H
atom	1.995134354	2.851376057	3.027721643	H
atom	5.094319344	7.325526237	12.829952240	H
atom	1.162436366	4.099842548	3.594649315	H
atom	5.927017212	8.573992729	12.263024330	H

Supplementary Table 9 | Input geometry.in file of IrBiSe corresponding to Supplementary Figure 19a.

lattice_vector	6.2899999619	0.0000000000	0.0000000000
lattice_vector	0.0000000000	6.2899999619	0.0000000000
lattice_vector	0.0000000000	0.0000000000	6.2899999619

atom	0.118063301	0.118063301	0.118063301	Ir
atom	3.026936531	6.171936989	3.263063192	Ir
atom	6.171936989	3.263063192	3.026936531	Ir
atom	3.263063192	3.026936531	6.171936989	Ir
atom	2.347805262	2.347805262	2.347805262	Bi
atom	0.797194660	3.942194462	5.492805481	Bi
atom	3.942194462	5.492805481	0.797194660	Bi
atom	5.492805481	0.797194660	3.942194462	Bi
atom	3.892377853	3.892377853	3.892377853	Se
atom	5.542622089	2.397622108	0.747377515	Se
atom	2.397622108	0.747377515	5.542622089	Se
atom	0.747377515	5.542622089	2.397622108	Se

Supplementary Table 10 | Input geometry.in file of racemic-NPB corresponding to Supplementary Figure 13c.

```
lattice_vector 19.28075432 0.11893738 -0.16084042
lattice_vector -0.04762409 7.89735002 -0.08918111
lattice_vector -0.23010841 0.09728093 8.74579059
atom 9.50419428 4.05496176 4.24359949 Pb
atom 9.64272421 0.05922138 -0.08488327 Pb
atom 9.59239632 5.39234681 1.47323567 Br
atom 9.53672969 1.45488956 2.65888312 Br
atom 9.41476145 2.71984904 7.01820401 Br
atom 9.47021079 6.65525434 5.83257514 Br
atom 6.52149394 3.81494085 4.06968567 Br
atom 12.58016831 7.75534441 0.00947094 Br
atom 12.48632053 4.29657061 4.42128484 Br
atom 6.42730750 0.35625700 8.48162335 Br
atom 4.40821563 3.85574320 0.73673337 C
atom 14.69102453 7.89780291 3.34024015 C
atom 14.59864031 4.25601932 7.75403740 C
atom 4.31568737 0.21403522 5.15070565 C
atom 3.27213868 4.72212750 0.63097143 C
atom 15.86383652 0.88311382 3.51494869 C
atom 15.73478673 3.38970660 7.85971080 C
atom 3.14284943 7.22878310 4.97583965 C
atom 4.23385780 2.57908024 1.23457073 C
atom 14.88127221 6.61224806 2.87196530 C
atom 14.77287811 5.53266184 7.25611800 C
atom 4.12543663 1.49962548 5.61889054 C
atom 5.07400758 1.89272073 1.30132753 H
atom 14.04976239 5.91407219 2.82147933 H
atom 13.93265113 6.21892457 7.18940671 H
```

atom	4.95696006	2.19777967	5.66950677	H
atom	1.98446113	4.21656116	1.01888294	C
atom	17.15762994	0.38495911	3.13798403	C
atom	17.02242507	3.89539564	7.47180502	C
atom	1.84904170	7.72697815	5.35264286	C
atom	3.34950118	6.06021372	0.15942559	C
atom	15.76999919	2.23045475	3.95622985	C
atom	15.65752930	2.05157234	8.33112315	C
atom	3.23667826	5.88147116	4.53446710	C
atom	4.07553436	6.58833605	8.61025571	H
atom	14.80863787	2.65595222	4.24171878	H
atom	14.93152366	1.52332794	-0.11980104	H
atom	4.19803853	5.45596306	4.24898397	H
atom	2.22209780	6.84795373	0.06472885	C
atom	16.88765343	3.03386745	4.03304352	C
atom	16.78500100	1.26392981	8.42579201	C
atom	2.11900498	5.07809480	4.45752960	C
atom	2.08350207	7.96206419	8.43283007	H
atom	16.78391800	4.05754612	4.38820256	H
atom	16.92370453	0.14975879	0.05755403	H
atom	2.22279026	4.05445458	4.10227835	H
atom	0.96162594	6.34505153	0.44907637	C
atom	18.15420940	2.53812406	3.65952878	C
atom	18.04544892	1.76699296	8.04158146	C
atom	0.85244331	5.57383558	4.83103157	C
atom	0.07472381	6.97108355	0.37343426	H
atom	19.03340407	3.17645550	3.72151180	H
atom	18.93241205	1.14105106	8.11725838	H
atom	-0.02673061	4.93547241	4.76911257	H
atom	0.84960544	5.05808138	0.92218063	C

atom	18.28202067	1.24243108	3.21509361	C
atom	18.15736460	3.05399587	7.56854037	C
atom	0.72463688	6.86952198	5.27548435	C
atom	-0.11894621	4.65666234	1.22201614	H
atom	19.25535225	0.84641859	2.92366112	H
atom	19.12588373	3.45550716	7.26872210	H
atom	-0.24869610	7.26558062	5.56684509	H
atom	2.97223226	2.09707439	1.64173866	C
atom	16.14875086	6.13681925	2.47543173	C
atom	16.03443560	6.01474558	6.84883317	C
atom	2.85789546	1.97514289	6.01513426	C
atom	2.89361627	1.08912138	2.04767412	H
atom	16.23994851	5.12077600	2.09299281	H
atom	16.11296164	7.02266209	6.44278718	H
atom	2.76666540	2.99124572	6.39740843	H
atom	1.86211331	2.89485016	1.51484088	C
atom	17.24877542	6.95121645	2.58322148	C
atom	17.14463748	5.21709418	6.97578273	C
atom	1.75785786	1.16076890	5.90726162	C
atom	0.87325864	2.53353032	1.79238889	H
atom	18.24199545	6.59598916	2.31354079	H
atom	18.13345177	5.57851682	6.69821958	H
atom	0.76460345	1.51604804	6.17674417	H
atom	5.77081351	4.35602016	0.28984409	C
atom	13.36982639	0.49383717	3.86489262	C
atom	13.23610362	3.75568535	8.20109541	C
atom	5.63685274	7.61785135	4.62616400	C
atom	5.94884163	5.35583936	0.71205107	H
atom	13.17932371	1.48127496	3.41960631	H
atom	13.05804304	2.75588620	7.77886009	H

atom	5.82714028	6.63031256	5.07131195	H
atom	6.87044011	3.50288351	0.85491611	N
atom	12.23339224	7.51147803	3.23087935	N
atom	12.13632720	4.60881474	7.63631414	N
atom	6.77333869	0.59995804	5.26049998	N
atom	6.88892475	2.54699538	0.43810792	H
atom	12.22670874	6.56534939	3.66970500	H
atom	12.11767394	5.56450237	8.05352484	H
atom	6.77980976	1.54636746	4.82223558	H
atom	6.76590845	3.42426882	1.89735058	H
atom	12.33891923	7.40999883	2.19056758	H
atom	12.24080039	4.68790512	6.59395884	H
atom	6.66796502	0.70076682	6.30094731	H
atom	7.79427204	3.95342209	0.69878286	H
atom	11.30398632	7.95406965	3.37662839	H
atom	11.21253951	4.15792863	7.79212373	H
atom	7.70271023	0.15764655	5.11416317	H
atom	5.72020651	4.50563373	7.52061133	C
atom	13.18972535	0.57903782	5.37835962	C
atom	13.28689702	3.60594956	0.97035684	C
atom	5.81706760	7.53289175	3.11270077	C
atom	5.69411612	3.49268538	7.10508102	H
atom	13.18096198	7.47380861	5.72816328	H
atom	13.31297953	4.61886201	1.38597599	H
atom	5.82634397	0.63823621	2.76311055	H
atom	6.67944854	4.96563778	7.25176869	H
atom	12.22483214	1.03312764	5.63682828	H
atom	12.32768069	3.14589237	1.23922153	H
atom	6.78175276	7.07839196	2.85419779	H
atom	4.91578233	5.08363807	7.05492168	H

atom	13.98690064	1.17771954	5.83017990	H
atom	14.09135894	3.02791920	1.43593977	H
atom	5.01961974	6.93470662	2.66069117	H

Supplementary Table 11 | Input geometry.in file of R-NPB corresponding to Supplementary Figure 13b.

lattice_vector	-1.49555572	-0.00681572	19.20931905	
lattice_vector	-0.03297736	-7.78884213	-0.00527112	
lattice_vector	8.76195420	-0.03700259	-0.14106161	
atom	5.05055629	3.41686722	19.05457723	Pb
atom	2.26202737	7.33591757	0.02111090	Pb
atom	6.72110530	3.41384287	2.81330377	Br
atom	0.59122119	7.32534769	16.26240531	Br
atom	3.57562082	4.60692884	0.13528501	Br
atom	3.71355208	0.69909699	18.93704058	Br
atom	8.45078011	5.81448294	0.17845392	Br
atom	-1.15116912	1.94779084	18.89523319	Br
atom	4.63889331	4.18574907	16.13461805	Br
atom	2.64683983	0.30827118	2.93703525	Br
atom	1.29117040	3.44014834	2.73660738	N
atom	6.02108489	7.30568918	16.33934104	N
atom	1.61655079	3.79071425	1.81768772	H
atom	5.69874718	7.65771389	17.25877065	H
atom	0.24297616	3.41194101	2.71866438	H
atom	7.06900180	7.26857252	16.35722182	H
atom	1.65659859	2.46418539	2.81739198	H
atom	5.64740582	6.33297286	16.25723482	H
atom	1.61814435	2.87942678	16.31843898	N
atom	5.68934041	6.76555143	2.75642261	N
atom	1.38207777	1.86464855	16.30230317	H
atom	5.91688413	5.74881239	2.77117340	H
atom	1.38205262	3.24990178	17.25991953	H
atom	5.92845444	7.13533124	1.81542350	H

atom	2.64759933	3.02659145	16.21256070 H
atom	4.66117532	6.92120779	2.86260580 H
atom	1.28640177	3.20243774	13.85047198 C
atom	6.02383117	7.08252123	5.22479560 C
atom	1.17329217	3.99925124	5.17957191 C
atom	6.11067828	0.07825180	13.89189404 C
atom	1.79821546	4.33315163	3.83755127 C
atom	5.48862185	0.41562881	15.23437349 C
atom	1.49471945	5.34530343	3.53361272 H
atom	5.80068452	1.42475243	15.53969449 H
atom	2.14927661	3.66809643	11.58215275 C
atom	5.16505735	7.55240233	7.49379556 C
atom	0.88370039	3.65473033	15.24411030 C
atom	6.43030110	7.53323952	3.83173778 C
atom	1.18772887	4.69277927	15.41195651 H
atom	6.10203688	0.78517284	3.65998916 H
atom	1.18306597	4.99343098	6.21143730 C
atom	6.10925152	1.07381927	12.86132993 C
atom	-0.61128845	3.54303057	15.49747094 C
atom	7.92430196	7.40929978	3.57822421 C
atom	-0.93593769	2.49633765	15.52290963 H
atom	8.24012996	6.35994323	3.55134144 H
atom	-1.15396059	4.03353719	14.68589287 H
atom	8.43807444	0.10524491	4.38521521 H
atom	-0.88990262	4.01315680	16.44782869 H
atom	8.17388947	0.08950634	2.62323956 H
atom	0.61126657	4.67373252	7.48866035 C
atom	6.67833187	0.75094479	11.58370702 C
atom	1.91703406	4.10700014	12.93414660 C
atom	5.36796745	0.20230333	6.13711262 C

atom	3.32008636	4.24367134	3.84540844	C
atom	3.96604924	0.33909067	15.22639574	C
atom	3.63690848	3.21684001	4.05328119	H
atom	3.67347070	7.10414083	15.02238606	H
atom	3.74166359	4.90183438	4.61166910	H
atom	3.55011814	1.00185862	14.46101196	H
atom	3.72119096	4.52541348	2.86575692	H
atom	3.56733256	0.62292242	16.20641754	H
atom	2.32715935	5.42164480	13.28010438	C
atom	4.96898638	1.52084550	5.79296873	C
atom	2.16843951	5.79366599	14.28918777	H
atom	5.13081511	1.89286818	4.78438043	H
atom	0.61957668	2.76203264	5.44282454	C
atom	6.68699395	6.62563093	13.63228925	C
atom	0.61466353	1.98823718	4.67827453	H
atom	6.68542402	5.85083259	14.39583454	H
atom	0.06616715	3.38443641	7.70910679	C
atom	7.24567623	7.24631134	11.36683244	C
atom	-0.35437575	3.15632918	8.68869684	H
atom	7.66436027	7.01600564	10.38696360	H
atom	0.96279581	1.92538457	13.42458381	C
atom	6.33663231	5.80219855	5.64895135	C
atom	0.47967263	1.22438172	14.10636343	H
atom	6.81375457	5.09805250	4.96618419	H
atom	1.72685800	6.29137891	6.03324969	C
atom	5.57651506	2.37612569	13.04119898	C
atom	2.17840038	6.58196119	5.08638269	H
atom	5.12744293	2.66931523	13.98843962	H
atom	0.05322875	2.45291970	6.69929344	C
atom	7.25078881	6.31341211	12.37544309	C

atom	-0.39462216	1.47260580	6.85668488	H
atom	7.69044728	5.32959679	12.21679593	H
atom	3.11067799	5.82998525	11.02721965	C
atom	4.18905978	1.93276339	8.04644469	C
atom	3.54140002	6.50928325	10.29621868	H
atom	3.76415013	2.61469243	8.77840156	H
atom	1.79994411	2.34992985	11.20183618	C
atom	5.50321348	6.23081147	7.87231019	C
atom	1.98431297	2.03222865	10.17483090	H
atom	5.31618230	5.91331676	8.89889852	H
atom	2.73075036	4.56398058	10.65163588	C
atom	4.55828546	0.66308115	8.42028797	C
atom	2.86311599	4.23856579	9.62106761	H
atom	4.42323381	0.33742448	9.45042927	H
atom	1.68208235	7.22966118	7.03999360	C
atom	5.62934790	3.31532139	12.03569100	C
atom	2.07706927	0.42579138	6.86328715	H
atom	5.20981494	4.30370711	12.20842056	H
atom	0.56687731	5.66764991	8.49782612	C
atom	6.73116850	1.74575569	10.57582401	C
atom	0.12005742	5.40576976	9.45730984	H
atom	7.17572342	1.48133387	9.61598435	H
atom	1.08775837	6.92316340	8.28185511	C
atom	6.22103058	3.00538995	10.79342220	C
atom	1.05509020	7.67700754	9.06888815	H
atom	6.26009914	3.75995577	10.00737453	H
atom	2.92490204	6.25276768	12.35737105	C
atom	4.37834007	2.35575932	6.71685486	C
atom	3.24738527	7.24899833	12.65064706	H
atom	4.06427508	3.35507526	6.42495049	H

atom	1.23580838	1.49018140	12.11232673	C
atom	6.06001743	5.36754079	6.96062907	C
atom	0.97170660	0.47395802	11.83007424	H
atom	6.31550757	4.34873185	7.24148194	H

Supplementary Table 12 | Input geometry.in file of S-NPB corresponding to Supplementary Figure 13a.

```
lattice_vector   -1.30473266    0.00799337    19.22478634
lattice_vector   -0.05336867   -7.78810274    -0.00001155
lattice_vector    8.76022728   -0.05995493    -0.22263015
atom    1.50003325    0.36546718     9.50593812 Pb
atom    5.98829082    4.22849770     9.49653022 Pb
atom    1.70258061    0.36940363    12.47230089 Br
atom    5.78608346    4.23592853     6.53016465 Br
atom    1.10624420    7.39071586     6.58963852 Br
atom    6.42557760    3.45928448    12.41264112 Br
atom    3.43526691    5.74185315     9.81905105 Br
atom    4.07386910    1.84319306     9.18340664 Br
atom    7.33296797    6.94308129     9.59941380 Br
atom    0.19309142    3.09795210     9.40260747 Br
atom    5.03200861    0.34333408    12.22331687 N
atom    2.45658563    4.25592292     6.77895308 N
atom    5.34445073   -0.00988284    11.30093523 H
atom    2.13908767    3.90708419     7.70128140 H
atom    5.40913843    1.31505001    12.30137611 H
atom    2.09290384    5.23275105     6.70082186 H
atom    3.98408315    0.38328051    12.21558358 H
atom    3.50495152    4.28141400     6.78686365 H
atom    0.68726629    4.81846245    12.42260609 N
atom    6.80924040    0.88264800     6.57986929 N
atom    0.91314068    4.44734249    11.47885961 H
atom    6.57843367    0.51459224     7.52362101 H
atom    0.92632956    5.83260533    12.43646584 H
atom    6.58401363    1.89994597     6.56603435 H
```

atom	-0.34147674	4.67411702	12.53851040	H
atom	7.83591682	0.72429009	6.46383867	H
atom	5.59312937	7.23176407	13.31848564	C
atom	1.93669952	3.36328652	5.68373058	C
atom	5.27536003	6.22335128	13.01671771	H
atom	2.24063640	2.35062753	5.98553029	H
atom	1.42992922	4.04093780	13.48955528	C
atom	6.05595191	0.11532695	5.51298806	C
atom	1.12091105	3.00387203	13.32488176	H
atom	6.40418578	6.86224350	5.67764392	H
atom	2.92265567	4.14779348	13.22114294	C
atom	4.56484014	0.24255557	5.78149925	C
atom	3.19018311	3.67630208	12.26827173	H
atom	4.34431109	7.56278830	6.73435749	H
atom	3.47193342	3.65602370	14.02746998	H
atom	4.06222253	7.54652910	4.97515777	H
atom	3.25033771	5.19344461	13.19179794	H
atom	4.25148767	1.29258907	5.81097289	H
atom	1.04265465	4.49451704	14.88713693	C
atom	6.44927394	0.56362637	4.11539615	C
atom	4.98541123	7.57150000	14.66692203	C
atom	2.54910175	3.69466070	4.33533790	C
atom	7.11591642	7.30421951	13.31146772	C
atom	0.41504449	3.45654897	5.69060639	C
atom	7.50418629	7.01889348	12.32768514	H
atom	0.02281268	3.17654266	6.67434443	H
atom	7.53753865	6.64067878	14.07304690	H
atom	-0.01555436	2.79884065	4.92898602	H
atom	7.39282846	0.53914908	13.51702978	H
atom	0.09880193	4.48399755	5.48501775	H

atom	4.99516638	6.57671527	15.69822610 C
atom	2.52581261	2.70005496	3.30407913 C
atom	0.20163116	4.03136896	17.16418731 C
atom	7.28407596	0.08931857	1.83836016 C
atom	0.41885423	3.59177915	15.80992015 C
atom	7.11409621	7.44065826	3.19254958 C
atom	1.37421989	5.77066347	15.30962006 C
atom	6.13506072	1.84419058	3.69300984 C
atom	1.85230515	6.47027908	14.62287814 H
atom	5.66649962	2.55016817	4.37980470 H
atom	4.39468229	1.02643953	14.93652668 C
atom	3.10339059	4.93023298	4.06568291 C
atom	4.39025741	1.80065729	14.17240825 H
atom	3.11838002	5.70434423	4.82977645 H
atom	0.00163517	2.27824309	15.46827344 C
atom	7.51319260	6.12145556	3.53402688 C
atom	0.14904141	1.90569908	14.45766999 H
atom	7.36051298	5.75079074	4.54453837 H
atom	5.52238408	5.27281420	15.51367497 C
atom	1.98086666	1.40347870	3.48870243 C
atom	5.95995543	4.97750782	14.56170805 H
atom	1.53931974	1.11421503	4.44068721 H
atom	4.44103212	6.90226982	16.98174134 C
atom	3.08433822	3.01794456	2.02055080 C
atom	5.47868784	4.33483330	16.52076586 C
atom	2.01174579	0.46493423	2.48166478 C
atom	5.89377385	3.34521933	16.34443092 H
atom	1.63653614	7.26919298	2.65804863 H
atom	-0.75993503	1.87209216	17.72906488 C
atom	8.26945386	5.70528467	1.27327004 C

atom	-1.18505995	1.19396897	18.46443617 H
atom	8.68536895	5.02152443	0.53785392 H
atom	3.84510278	1.34114748	16.19905009 C
atom	3.65723306	5.23734144	2.80314559 C
atom	3.40970731	2.32624144	16.36151536 H
atom	4.10605463	6.21638574	2.64064912 H
atom	3.85889282	0.40916957	17.20842975 C
atom	3.63073344	4.30560414	1.79379663 C
atom	3.45154459	0.64139410	18.19262358 H
atom	4.04122116	4.53221982	0.80959948 H
atom	1.11596913	6.20665378	16.62460799 C
atom	6.39929701	2.27676236	2.37808214 C
atom	1.38596125	7.22209735	16.90410319 H
atom	6.14312635	3.29580212	2.09866879 H
atom	-0.37282786	3.13711228	18.10064142 C
atom	7.89975210	6.97554315	0.90184719 C
atom	-0.49383920	3.46298149	19.13246427 H
atom	8.02535523	7.29991102	-0.12989882 H
atom	0.55868015	5.34852084	17.54084733 C
atom	6.94502718	1.41125636	1.46181570 C
atom	0.38583866	5.66682407	18.56967211 H
atom	7.12230969	1.72729226	0.43304802 H
atom	-0.58884033	1.44872904	16.39712249 C
atom	8.09234339	5.28406867	2.60510660 C
atom	-0.91688469	0.45332341	16.10720905 H
atom	8.40662478	4.28419311	2.89487993 H
atom	4.39722299	5.90867968	17.99127130 C
atom	3.11458592	2.02379430	1.01107388 C
atom	3.96411666	6.17522146	18.95575892 H
atom	3.55128897	2.28433982	0.04657407 H

atom	4.90200716	4.64762409	17.76937198	C
atom	2.59263159	0.76975868	1.23303592	C
atom	4.87026043	3.89410491	18.55675819	H
atom	2.61408678	0.01582451	0.44569902	H

Supplementary Table 13 | Input geometry.in file of S-MBPI corresponding to Supplementary Figure 16.

lattice_vector	-0.00114963	0.01873775	28.82929235	
lattice_vector	8.80819805	-0.00043968	-0.00207826	
lattice_vector	0.00055913	9.18552508	-0.00126745	
atom	5.31907260	0.06237008	0.03964829	Pb
atom	7.89340709	9.11360039	14.45392082	Pb
atom	3.48918573	4.66497955	14.37615526	Pb
atom	0.91395803	4.53042484	28.79326708	Pb
atom	3.73065964	2.94992950	28.80628490	I
atom	0.67250853	6.24575994	14.38963528	I
atom	5.07682068	7.53316930	14.44175324	I
atom	8.13582409	1.64280388	0.02537187	I
atom	2.59450395	7.40943394	0.00765125	I
atom	1.81007891	1.76709793	14.42302549	I
atom	6.21432680	2.82643768	14.40842563	I
atom	6.99749753	6.36903489	28.82418036	I
atom	5.47200934	8.91382496	3.23363892	I
atom	7.74048247	0.26929687	17.64940543	I
atom	3.33617560	4.32364180	11.18161780	I
atom	1.06712216	4.86463418	25.59841359	I
atom	5.51377854	8.92348237	25.65810670	I
atom	7.69829372	0.26358135	11.24460167	I
atom	3.29449321	4.32902076	17.58635334	I
atom	1.10998590	4.85453368	3.17385677	I
atom	8.46602159	9.06474630	21.60644866	C
atom	4.74685038	0.12031824	7.19463300	C
atom	0.34258177	4.47258291	21.63906933	C
atom	4.06113635	4.71326140	7.22300822	C

atom	0.66140898	8.21118859	21.16037769	C
atom	3.74353171	0.97430759	6.74568034	C
atom	8.14716255	3.61839744	22.08569648	C
atom	5.06454889	5.56735199	7.67142300	C
atom	0.59151034	8.99389635	23.84425385	C
atom	3.81218160	0.19206006	9.42998546	C
atom	8.21672087	4.40026643	19.40140055	C
atom	4.99568353	4.78409999	4.98761986	C
atom	8.42359803	0.25417964	22.94796796	C
atom	4.78862292	8.93110430	8.53371624	C
atom	0.38426849	4.84712289	20.29866481	C
atom	4.01980402	4.33803518	5.88283727	C
atom	1.63086686	7.74482993	22.04669923	C
atom	2.77370162	1.44076298	7.63141848	C
atom	7.17750227	3.15194183	21.19972046	C
atom	6.03435221	6.03347344	6.78540771	C
atom	1.60190856	8.13982384	23.38109628	C
atom	2.80199728	1.04595732	8.96596472	C
atom	7.20626997	3.54654485	19.86514543	C
atom	6.00593358	5.63818558	5.45106924	C
atom	5.41531845	5.33610722	23.69531024	C
atom	7.79663332	3.85007573	9.27945905	C
atom	3.39264687	0.74302860	19.55113705	C
atom	1.01161877	8.44243847	5.13721388	C
atom	5.86299163	3.52133634	25.43231280	C
atom	7.34832830	5.66458250	11.01662555	C
atom	2.94512009	8.11330090	17.81369899	C
atom	1.45933441	1.07076637	3.40043302	C
atom	5.70048152	5.00631746	25.14479322	C
atom	7.51149709	4.17977886	10.72885930	C

atom	3.10751623	0.41279145	18.10184492	C
atom	1.29680911	8.77156800	3.68759768	C
atom	6.30138438	6.15804737	22.98873521	C
atom	6.91073071	3.02794629	8.57296462	C
atom	2.50647400	1.56505472	20.25737931	C
atom	1.89733857	7.62032783	5.84405647	C
atom	4.32731085	4.77075034	23.01733407	C
atom	0.07651005	4.41582505	8.60363566	C
atom	4.48070687	0.17796927	20.22932422	C
atom	8.73193511	9.00788913	5.81283511	C
atom	6.10637280	6.40679376	21.63193821	C
atom	7.10601463	2.77895779	7.21628799	C
atom	2.70134767	1.81408031	21.61411463	C
atom	1.70199889	7.37187358	7.20084699	C
atom	4.13633106	5.01736143	21.65916715	C
atom	0.26777409	4.16894832	7.24559849	C
atom	4.67153898	0.42484224	21.58743042	C
atom	8.54054549	8.76144334	7.17096184	C
atom	5.02367946	5.83887065	20.96613911	C
atom	8.18881815	3.34679965	6.55056730	C
atom	3.78401301	1.24634342	22.28016129	C
atom	0.61926935	7.94011783	7.86631331	C
atom	0.55983906	0.29641337	25.27559328	C
atom	3.84383755	8.89056018	10.85907369	C
atom	8.24783685	4.88760335	17.97107734	C
atom	4.96466063	4.29625366	3.55755754	C
atom	1.86754709	0.88565315	25.77004636	C
atom	2.53612835	8.30254777	11.35497538	C
atom	6.94018336	5.47648398	17.47597973	C
atom	6.27232292	3.70742973	3.06255367	C

atom	7.73298008	0.27063880	20.90326377	H
atom	5.48017391	8.91423371	6.48943918	H
atom	1.07522061	4.86412154	22.34322428	H
atom	3.32840070	4.32198148	7.92721975	H
atom	0.68896782	7.93394747	20.10781472	H
atom	3.71644884	1.25150613	5.69307920	H
atom	8.11971634	3.34141481	23.13835526	H
atom	5.09169345	5.84487534	8.72391307	H
atom	7.63043187	0.90754953	23.30954467	H
atom	5.58205803	8.27804769	8.89537372	H
atom	1.17763070	5.50016691	19.93684440	H
atom	3.22652391	3.68481640	5.52122011	H
atom	2.44096063	7.10709636	21.69745599	H
atom	1.96380482	2.07841751	7.28149929	H
atom	6.36741298	2.51441265	21.54940259	H
atom	6.84432112	6.67122456	7.13492317	H
atom	2.39427924	7.80058559	24.04929612	H
atom	2.00919836	1.38521999	9.63361368	H
atom	6.41367500	3.20727604	19.19724965	H
atom	6.79864146	5.97720353	4.78316649	H
atom	3.65808414	5.24922782	25.81630958	H
atom	0.74606455	3.93826933	11.40196267	H
atom	5.15002266	0.65442958	17.42993780	H
atom	8.06224059	8.52817006	3.01511161	H
atom	4.64524484	6.62279398	25.98055979	H
atom	8.56776368	2.56376738	11.56428252	H
atom	4.16361266	2.02860949	17.26596929	H
atom	0.24118375	7.15513067	2.85209950	H
atom	4.80848763	5.33098745	27.03350928	H
atom	8.40396684	3.85505253	12.61737290	H

atom	3.99909249	0.73732590	16.21299609	H
atom	0.40359948	8.44718502	1.79927961	H
atom	6.19331465	3.34690640	26.46430708	H
atom	7.01830349	5.83852228	12.04877580	H
atom	2.61486946	7.93859619	16.78174665	H
atom	1.78947093	1.24432232	2.36823742	H
atom	6.62444593	3.10866595	24.76272101	H
atom	6.58645521	6.07704571	10.34733920	H
atom	2.18350971	7.70115020	18.48346882	H
atom	2.22115048	1.48356918	4.06953472	H
atom	4.93305411	2.96218102	25.27106154	H
atom	8.27795468	6.22421913	10.85522762	H
atom	3.87489901	7.55396641	17.97529127	H
atom	0.52968438	1.63046012	3.56146810	H
atom	6.61554671	5.53303205	25.44941172	H
atom	6.59681867	3.65252284	11.03371738	H
atom	2.19267070	0.93971527	17.79688219	H
atom	2.21156967	8.24429132	3.38307065	H
atom	7.16963271	6.58252786	23.49372562	H
atom	6.04242008	2.60349828	9.07790257	H
atom	1.63825483	1.98936830	19.75215593	H
atom	2.76556435	7.19550286	5.33933092	H
atom	3.62823968	4.11949104	23.54325510	H
atom	0.77550128	5.06714259	9.12953283	H
atom	5.18049732	8.71235562	19.70229807	H
atom	8.03290371	0.47410139	5.28794602	H
atom	6.82773577	7.01712616	21.09262486	H
atom	6.38477823	2.16851206	6.67697788	H
atom	1.97988071	2.42447831	22.15314955	H
atom	2.42309790	6.76152643	7.74051589	H

atom	3.31719724	4.53694897	21.12748250	H
atom	1.08698921	4.64928637	6.71395224	H
atom	5.49129579	9.13014897	22.11799821	H
atom	7.72097047	0.05665552	7.70373998	H
atom	4.87707692	6.00346594	19.89960455	H
atom	8.33569150	3.18200835	5.48412475	H
atom	3.93050525	1.41102481	23.34667914	H
atom	0.47236315	7.77557672	8.93280894	H
atom	8.57229159	1.04743269	25.37631303	H
atom	4.63919816	8.13872542	10.96235152	H
atom	0.23533050	5.63952736	17.86994779	H
atom	4.16903144	3.54468635	3.45462032	H
atom	0.57014055	7.43871610	25.97882228	H
atom	3.83279017	1.74814126	11.56517415	H
atom	8.23697032	2.84448824	17.26634500	H
atom	4.97465451	6.33945098	2.85287448	H
atom	7.88767584	8.23218651	26.08538885	H
atom	5.32478596	0.95601293	11.66971446	H
atom	0.92041095	3.63741436	17.16194304	H
atom	3.48349604	5.54624481	2.74587987	H
atom	0.29919538	8.58514948	27.17859911	H
atom	4.10897400	0.60119978	12.76396107	H
atom	8.51112952	3.99135273	16.06716508	H
atom	4.70169617	5.19295743	1.65346513	H
atom	2.69605275	0.17007806	25.72791172	H
atom	1.70794602	9.01847942	11.31270145	H
atom	6.11168716	4.76090773	17.51827870	H
atom	7.10073490	4.42310377	3.10468445	H
atom	1.77417124	1.24805437	26.80080535	H
atom	2.63014592	7.94136639	12.38612662	H

atom	7.03382870	5.83820158	16.44498013	H
atom	6.17860212	3.34546093	2.03167592	H
atom	2.12603012	1.74552239	25.14145072	H
atom	2.27668123	7.44201025	10.72777124	H
atom	6.68144217	6.33677166	18.10381583	H
atom	6.53113995	2.84730578	3.69066695	H
atom	4.62284930	5.57650841	26.04641414	N
atom	8.58970243	3.61004807	11.63021058	N
atom	4.18539917	0.98232586	17.20006528	N
atom	0.21870477	8.20142333	2.78643485	N
atom	0.11480264	8.35344803	26.18796542	N
atom	4.28985898	0.83400967	11.77288115	N
atom	8.69352082	3.75875747	17.05802123	N
atom	4.51865052	5.42494699	2.64427730	N

Supplementary References

1 Smith, M. D., Jaffe, A., Dohner, E. R., Lindenberg, A. M. & Karunadasa, H. I. Structural origins of broadband emission from layered Pb–Br hybrid perovskites. *Chem. Sci.* **8**, 4497-4504 (2017).

2 Shibuya, K., Koshimizu, M., Nishikido, F., Saito, H. & Kishimoto, S. Poly[bis(phenethylammonium) [dibromidoplumbate(II)]-di-[mu]-bromido]]. *Acta Cryst.* **65**, m1323-m1324 (2009).

3 Du, K.-z. *et al.* Two-Dimensional Lead(II) Halide-Based Hybrid Perovskites Templated by Acene Alkylamines: Crystal Structures, Optical Properties, and Piezoelectricity. *Inorg. Chem.* **56**, 9291-9302 (2017).

4 Mao, L. *et al.* Structural Diversity in White-Light-Emitting Hybrid Lead Bromide Perovskites. *J. Am. Chem. Soc.* **140**, 13078-13088 (2018).

5 Mao, L., Wu, Y., Stoumpos, C. C., Wasielewski, M. R. & Kanatzidis, M. G. White-Light Emission and Structural Distortion in New Corrugated Two-Dimensional Lead Bromide Perovskites. *J. Am. Chem. Soc.* **139**, 5210-5215 (2017).

6 Kepenekian, M. *et al.* Rashba and Dresselhaus Effects in Hybrid Organic–Inorganic Perovskites: From Basics to Devices. *ACS Nano* **9**, 11557-11567 (2015).

7 FermiLoop: Calculation on a constant-energy level. http://www.openmx-square.org/openmx_man3.9/node176.html (2018).

8 Kotaka, H., Ishii, F. & Saito, M. Rashba Effect on the Structure of the Bi One-Bilayer Film: Fully Relativistic First-Principles Calculation. *Jpn. J. Appl. Phys.* **52**, 035204 (2013).

9 Ozaki, T. & Kino, H. Efficient projector expansion for the ab initio LCAO method. *Phys. Rev. B* **72**, 045121 (2005).

10 Schmitt, T. *et al.* Control of Crystal Symmetry Breaking with Halogen-Substituted Benzylammonium in Layered Hybrid Metal-Halide Perovskites. *J. Am. Chem. Soc.* **142**, 5060-5067 (2020).

11 Liu, Z. *et al.* A Giant Bulk-Type Dresselhaus Splitting with 3D Chiral Spin Texture in IrBiSe. *Phys. Status Solidi RRL* **14**, 1900684 (2020).
